# Glycosylated nanoparticle-based PfCSP vaccine confers long-lasting antibody responses and sterile protection in mouse malaria model

**DOI:** 10.1038/s41541-023-00653-7

**Published:** 2023-04-07

**Authors:** Julia Ludwig, Stephen W. Scally, Giulia Costa, Sandro Hoffmann, Rajagopal Murugan, Jana Lossin, Katherine Prieto, Anna Obraztsova, Nina Lobeto, Blandine Franke-Fayard, Chris J. Janse, Celia Lebas, Nicolas Collin, Spela Binter, Paul Kellam, Elena A. Levashina, Hedda Wardemann, Jean-Philippe Julien

**Affiliations:** 1 B Cell Immunology, German Cancer Research Center (DKFZ), Heidelberg, Germany; 2 Program in Molecular Medicine, The Hospital for Sick Children Research Institute, Toronto, ON Canada; 3 Department of Immunology, University of Toronto, Toronto, ON Canada; 4 Vector Biology Unit, Max Planck Institute for Infection Biology, Berlin, Germany; 5 Malaria Research Group, Department of Parasitology, Leiden University Medical Center, Leiden, The Netherlands; 6 Vaccine Formulation Institute, Plan-les-Ouates, Switzerland; 7 Kymab a Sanofi Company, Babraham Research Campus, Cambridge, UK; 8 Department of Infectious Diseases, Faculty of Medicine, Imperial College London, London, UK; 9 Department of Biochemistry, University of Toronto, Toronto, ON Canada

**Keywords:** Malaria, Protein vaccines

## Abstract

The development of an effective and durable vaccine remains a central goal in the fight against malaria. Circumsporozoite protein (CSP) is the major surface protein of sporozoites and the target of the only licensed *Plasmodium falciparum* (Pf) malaria vaccine, RTS,S/AS01. However, vaccine efficacy is low and short-lived, highlighting the need for a second-generation vaccine with superior efficacy and durability. Here, we report a *Helicobacter pylori* apoferritin-based nanoparticle immunogen that elicits strong B cell responses against PfCSP epitopes that are targeted by the most potent human monoclonal antibodies. Glycan engineering of the scaffold and fusion of an exogenous T cell epitope enhanced the anti-PfCSP B cell response eliciting strong, long-lived and protective humoral immunity in mice. Our study highlights the power of rational vaccine design to generate a highly efficacious second-generation anti-infective malaria vaccine candidate and provides the basis for its further development.

## Introduction

Malaria caused by the unicellular parasite *Plasmodium falciparum* (Pf) remains a major health problem particularly in sub-Saharan Africa, which carries nearly 95% of the global disease burden^1^. The only available malaria vaccine, RTS,S/AS01 (Mosquirix, GSK Biologicals, short RTS,S), targets the pre-erythrocytic sporozoite stage of the parasite that is transmitted to humans by the bite of infected *Anopheles* mosquitoes. RTS,S has been developed more than 30 years ago with the aim to prevent the infection through the induction of antibody responses against Pf circumsporozoite protein (PfCSP) that densely coats the surface of sporozoites and is essential for parasite development^2–5^. PfCSP contains three domains: the N-terminal domain (N-CSP), the central repeat domain and the C-terminal domain (C-CSP), which anchors the protein to the cell membrane through a GPI-linker^6,7^. To induce protective humoral immunity, RTS,S includes part of the PfCSP central repeat domain comprising 18 repeating motifs composed of asparagine, alanine, asparagine and proline (NANP)^8,9^ that are known targets of potent antibodies and highly conserved across Pf isolates^10^. In contrast, the vast majority of reported PfCSP T cell epitopes are located in the polymorphic C-CSP^11–18^. To provide T cell help, RTS,S contains the complete C-CSP of the Pf laboratory strain NF54^8^. Immunogenicity is further boosted through genetic fusion with the Hepatitis B surface antigen (HBsAg) and complexing of the recombinant fusion protein with free HBsAg (S) for self-assembly into virus-like particles.

Vaccination with RTS,S in a monophosphoryl lipid A and saponin containing adjuvant system (AS01), induces strong humoral anti-NANP responses in humans. However, a large phase III field trial and recent implementation studies have shown that its efficacy is overall limited and protection is relatively short-lived^19^, which might be related to a drop in anti-PfCSP-reactive IgG and IgM antibodies at around 6 months after vaccination^20^. However, the exact reasons for this remain elusive and it remains to be determined whether the Matrix M saponin-adjuvanted R21, an evolved immunogen version that lacks free HBsAg and, therefore, has an increased proportion of PfCSP components compared to RTS,S, will provide better efficacy and longevity in phase III trials^21–23^. To instruct the design of a second-generation PfCSP vaccine, recent studies have dissected the human anti-PfCSP antibody response at monoclonal level^10,24–31^. The work aimed at defining the target epitopes of the most potent anti-PfCSP antibodies beyond the NANP repeats and to characterize the molecular and cellular mechanisms that drive the induction of protective humoral anti-PfCSP immunity. To date no potent antibody targeting the N-CSP or C-CSP could be identified, suggesting that these regions are poor vaccine targets^25,29,32,33^. In contrast, several additional target epitopes of potent antibodies were discovered in the junction that links the N-CSP and the NANP central repeats by a short stretch of a few alternating NANP and NANP-like NPDP and NVDP motifs (junction) that is absent from RTS,S^26,30,31,34^. Due to the high degree of similarity between these minor motifs in the junction and the NANP repeats, most anti-junction antibodies show cross-reactivity with the NANP-repeat motifs^26,30,31^. Cross-reactivity was linked to antibody binding strength and parasite inhibition suggesting that inclusion of the junction might promote the induction of potent humoral anti-parasite responses.

Here, we integrated our combined molecular and cellular understanding of anti-PfCSP antibodies and B cell affinity maturation to design a PfCSP-based immunogen that induced strong long-lasting parasite-inhibitory serum-antibody responses and protection in mice, laying the foundational basis for its further development.

## Results

### Antibody responses against the NANP repeats but not the immunodominant C-CSP show parasite-inhibitory activity

**Fig. 1:**
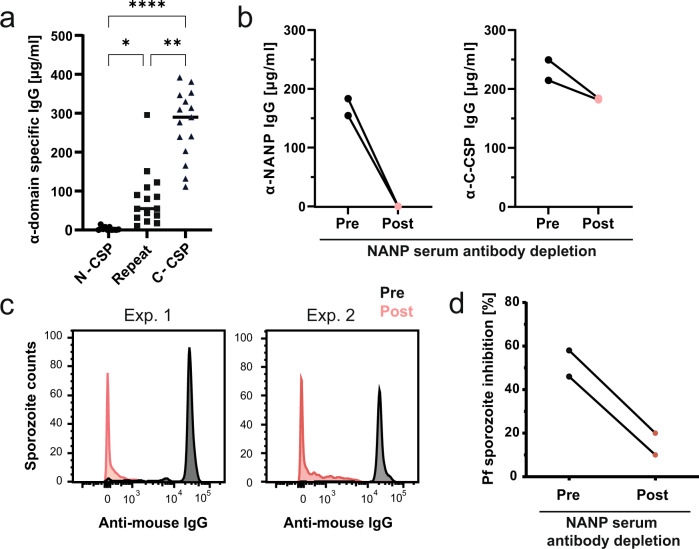
The C-CSP of recombinant PfCSP is highly immunogenic but fails to induce protective serum-antibody responses in mice. **a** C57BL/6J mice were immunized with FL-PfCSP at day 0, 21 and 42 adjuvanted with SAS. The serum IgG concentration against the indicated PfCSP domains was determined 7 days after the last immunization. Dots represent data from individual mice. Pooled data of three independent experiments with 5 mice per group are shown. Black horizontal lines indicate arithmetic means. Statistically significant differences were calculated by Kruskal–Wallis test with Dunn’s correction (*P < 0.05; **P < 0.01; ****P < 0.0001). **b** Sera, collected 7 days after the last immunization, from 5 mice of the same experimental group were pooled and NANP-reactive antibodies were depleted. The IgG concentration of NANP and C-CSP reactive antibodies was determined before (pre) and after (post) depletion. Dots represent independent experiments. **c** Binding profile to Pf sporozoites of the pooled sera described in (**b**) diluted 1:100. Gating on Pf sporozoites is shown in Supplementary Fig. 1. **d** Capacity of the pooled sera described in (**c**) to inhibit the hepatocyte traversal activity of Pf sporozoites in vitro. Dots represent independent traversal assay experiments. Gating strategy for Pf traversal analysis is shown in Supplementary Fig. 2.

To determine whether the C-CSP induces protective humoral immune responses and may play an important role in vaccine design beyond inducing T cell help, we directly compared the protective properties of antibodies targeting the different PfCSP domains. Repeated immunization of C57BL/6J wild type (wt) mice with a recombinant full-length PfCSP (FL-PfCSP) adjuvanted with SAS, an oil-in-water adjuvant system containing MPL in combination with trehalose dicorynomycolate (TDM)^35^, induced anti-PfCSP IgG responses dominated by antibodies against the C-CSP compared to NANP repeats, whereas antibodies against the N-CSP were barely detectable (Fig. 1a). Thus, in contrast to the known immunodominance of the NANP repeat region in anti-PfCSP antibody responses induced by sporozoites^24,34,36,37^, the C-CSP is highly immunodominant in recombinant soluble FL-PfCSP.

Given the overall low immunogenicity and lack of evidence of potent antibody epitopes in the N-CSP^25^, we focused our further analyses on the NANP repeats and C-CSP. To dissect the relative contribution of antibodies against NANP repeats and C-CSP to parasite inhibition, we depleted the immune sera of NANP-reactive antibodies by affinity purification and compared serum-antibody binding to live Pf sporozoites before and after the depletion by flow cytometry (Fig. 1b, c). Only sera containing NANP-reactive IgG antibodies showed sporozoite binding to Pf sporozoites, suggesting that the C-CSP-reactive antibodies failed to access their target epitopes on the surface of live sporozoites (Fig. 1c, Supplementary Fig. 1). In line with the lack of sporozoite binding, NANP-antibody depletion also strongly reduced sera potency to inhibit Pf sporozoite traversal of hepatocytes in vitro (Fig. 1d, Supplementary Fig. 2). Thus, despite the high immunogenicity of the C-CSP and strong reactivity of the antibodies with recombinant PfCSP, this component of the response lacked direct functional relevance. Instead, the parasite inhibitory activity of humoral immune responses against PfCSP was linked primarily to antibodies against the central repeat region.

### *Helicobacter pylori* apoferritin-based PfCSP nanocage immunogens induce strong parasite-inhibitory antibody responses

**Fig. 2:**
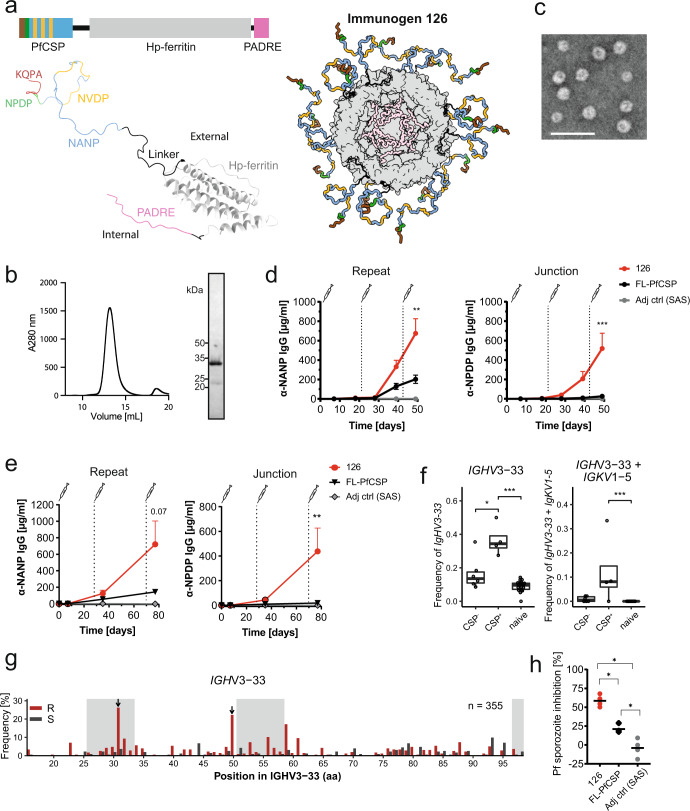
Design and immunogenicity of a PfCSP nanoparticle immunogen. **a** Schematic representation of immunogen 126, comprising the PfCSP epitope motifs KQPADG (brown), NPDP (green), NANP (blue) and NVDP (yellow) genetically fused to *H. pylori* apoferritin (gray) and PADRE (pink), separated by short linkers (black). Models of immunogen 126 displayed as both a monomer and as an assembled nanoparticle. The PfCSP epitope is presented externally on the nanoparticle surface. For better illustration, six monomers have been removed from the assembled nanoparticle, thus slicing through the immunogen, to reveal the PADRE epitopes within its core. **b** Size exclusion chromatogram and SDS-PAGE analysis of immunogen 126 (uncropped SDS-PAGE in Supplementary Fig. 3a). **c** Negative stain electron microscopy of immunogen 126. Scale bar—50 nm. **d** C57BL/6J mice were immunized with FL-PfCSP or immunogen 126 adjuvanted with SAS at day 0, 21 and 42. The serum IgG response against the PfCSP repeat (NANP) and junction (NPDP) was determined at the indicated timepoints. Immunization with SAS alone served as negative control. Pooled data of two independent experiments with 5 mice per group are shown. **e** Mice from the Kymouse™ platform were immunized with FL-PfCSP or immunogen 126 adjuvanted with SAS at day 0, 28 and 70. The serum IgG response against the PfCSP repeat (NANP) and the junction (NPDP) was measured at the indicated timepoints. Immunization with SAS alone served as control. One representative out of two independent experiments with 7 mice per group is shown. **f** *IGHV3-33* (left) and paired *IGHV3-33*/*IGKV1-5* (right) gene usage frequency among sorted GC CSP+ or GC CSP− cells isolated from lymph nodes of mice from the Kymouse™ platform 7 days after the third immunization with immunogen 126 adjuvanted with SAS. Data from naïve B cells are shown for comparison. FACS gating strategy is shown in Supplementary Fig. 6. Dots represent individual mice. Pooled data of two independent experiments are shown (CSP−: *n* = 6, CSP+: *n* = 4; naïve: *n* = 20). **g** Silent (S, gray) and replacement (red) mutations in VH3-33 antibodies of mice from the Kymouse™ platform. CDRs are marked in light gray. Arrows indicate positions H.31 and H.50 with strong selection for replacement mutations. **h** Capacity of pooled sera (diluted 1:400, collected 7 days after the last immunization) from mice of the Kymouse™ platform to inhibit the hepatocyte traversal activity of Pf sporozoites in vitro. Dots represent independent traversal assay experiments (*n* = 4). Arithmetic mean (**d**, **e**, **h**), median with length of the whiskers as multiple of IQR (**f**) and SEM (**d**, **e**) are indicated. Statistically significant differences were calculated by two-tailed Mann–Whitney test. In (**d**) and (**e**) statistical analyses were performed with data from day 50 and day 80, respectively (*P < 0.05; **P < 0.01; ***P < 0.001). Statistically non-significant differences are not indicated.

To avoid the induction of strong antibody responses against the C-CSP, we focused our immunogen design on the NANP repeat and the junction, the target epitopes of high-affinity, cross-reactive and protective antibodies^10,24,26,28,30,31^. Furthermore, we limited the number of repeating NANP motifs to five per PfCSP peptide to avoid the potential activation of low-affinity B cells by strong BCR cross-linking^34,38–40^. To compensate for the lack of PfCSP-derived T cell epitopes in the absence of the C-CSP and to promote affinity maturation, we used a universal pan DR T helper cell epitope PADRE known to induce a strong response in humans and mice^41^. The PfCSP and PADRE sequences were genetically fused to the N- and C-terminus, respectively, of *H. pylori* apoferritin, a self-assembling protein that oligomerizes into spherical nanocages with 24 subunits (Fig. 2a). N-terminal fusion of the PfCSP peptides enabled their display on the particle surface to mediate efficient B cell activation, whereas the C-terminal PADRE peptides were encapsulated inside the nanocage, thereby avoiding the induction of humoral immune responses against these T cell epitopes. This nanoparticle, referred to as immunogen 126, assembled into monodisperse, well-formed spherical particles (Fig. 2b, c), and bound strongly to monoclonal antibodies (mAbs) reactive to the junction and NANP repeats, but not N- or C-CSP (Supplementary Fig. 4a).

Immunization of wt mice with immunogen 126 adjuvanted with SAS induced a long-lasting and significantly stronger antibody response against the junction and NANP repeats compared to immunization with the FL-PfCSP monomer (Fig. 2d and Supplementary Fig. 5). Similar results were obtained in mice of the Kymouse™ platform transgenic for the entire human variable gene repertoire^42^ (Fig. 2e). Ig gene sequencing of PfCSP-reactive B cells from these transgenic mice immunized with immunogen 126 adjuvanted with SAS showed that the response was dominated by VH3-33 antibodies paired with VK1-5 light chains, a combination associated with high anti-PfCSP antibody reactivity in humans^24^ (Fig. 2f, Supplementary Fig. 6). The vast majority of VH3-33 antibodies carried somatic mutations and showed clear signs of affinity maturation based on the selection of somatic hypermutations previously shown to increase PfCSP affinity (H.31, H.50)^24,43^ (Fig. 2g), demonstrating the efficiency of the employed immunogen design strategy.

The potency of immunogen 126 was also reflected by the parasite inhibitory activity of the elicited humoral immune response. Sera from mice of the Kymouse™ platform immunized with immunogen 126 were significantly more efficient at blocking Pf sporozoite traversal of hepatocytes than sera from FL-PfCSP immunized animals with overall lower titers and lack of junction-reactive antibody responses (Fig. 2h). In summary, immunization with the newly designed nanoparticle immunogen 126 induced high and stable antibody responses against the protective PfCSP epitopes in the junction and NANP repeats that showed clear signs of efficient affinity maturation and mediated strong parasite inhibitory activity.

### Glycan engineering of the nanocage scaffold focuses and enhances the humoral response against PfCSP epitopes

**Fig. 3:**
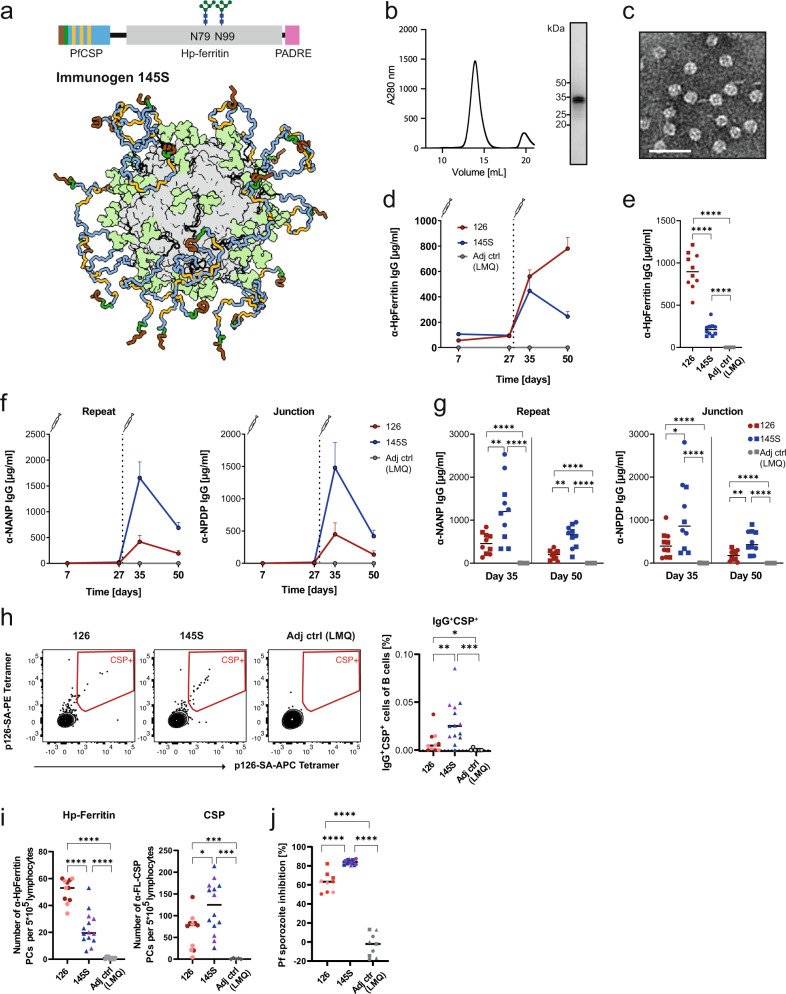
Glycan modifications of the nanoparticle focus the humoral response on PfCSP epitopes. **a** Schematic representation of immunogen 145S. Immunogen 126 (Fig. 2a) was modified by the engineering of two non-native N-linked glycosylation sites at positions N79 and N99 of *H. pylori* apoferritin. Expression of these nanoparticles in HEK293S (GnT I^−/−^) cells led to an addition of high mannose sugars at the targeted positions. This glycosylation (colored light green) covers the *H. pylori* apoferritin nanoparticle surface while not affecting presentation of the PfCSP epitope (scheme below). **b** Size exclusion chromatogram and SDS-PAGE analysis of immunogen 145S (uncropped SDS-PAGE in Supplementary Fig. 3b). **c** Negative stain electron microscopy of immunogen 145S. Scale bar—50 nm. **d** C57BL/6J mice were immunized with immunogen 126 or 145S adjuvanted with LMQ at day 0 and 28. The serum IgG response against *H. pylori* apoferritin was measured at various timepoints. Immunization with LMQ alone served as control. One representative out of two independent experiments with 5 mice per group is shown. **e** *H. pylori* apoferritin IgG response 22 days after the last immunization with immunogen 145S or 126 compared to adjuvant alone as negative control. Dots represent individual mice. Pooled data of two independent experiments with 5 mice per group are shown. Symbols indicate independent experiments. **f** Serum IgG response of the mice described in (**d**) against the PfCSP repeat (NANP) and the junction (NPDP). One representative out of two independent experiments with 5 mice per group is shown. **g** Comparison of the IgG response against the PfCSP repeat (NANP) and junction (NPDP) at 7 days and 22 days after the last immunization. Dots represent individual mice. Pooled data of two independent experiments with 5 mice per group are shown. Symbols indicate independent experiments. **h** Representative FACS analysis (left) and quantification (right) of live IgG+ antigen-binding cells in lymph nodes of mice immunized with immunogen 126, 145S or adjuvant alone 22 days after the last immunization. Dots represent individual mice. Pooled data of two independent experiments are shown. Symbols indicate independent experiments. Dark red (*n* = 6) and blue (*n* = 9): 10 µg dose immunization; light red (*n* = 5) and violet (*n* = 5): 0.5 µg dose immunization; adjuvant control group (*n* = 5). Detailed gating strategy is shown in Supplementary Fig. 9. **i** ELISpot-based enumeration of *H. pylori* apoferritin and FL-CSP reactive bone marrow plasma cells (PCs) of mice 22 days after the last immunization with immunogen 145S or 126 at 10 µg (dark red (*n* = 6) and blue (*n* = 9), respectively) or 0.5 µg (light red (*n* = 5) and violet (*n* = 5), respectively) or adjuvant alone (*n* = 10). Dots represent individual mice. Pooled data of two independent experiments are shown. Symbols indicate independent experiments. **j** Capacity of pooled sera (diluted 1:800, collected 22 days after the last immunization) from the same mice as in (**i**) to inhibit the hepatocyte traversal activity of Pf sporozoites in vitro. Pooled data of three independent traversal assay experiments are shown. Dark red (*n* = 6) and blue (*n* = 9): 10 µg dose; light red (*n* = 6) and violet (*n* = 6): 0.5 µg dose; adjuvant control group (*n* = 9). Symbols indicate independent immunization experiments. Arithmetic mean (**d**–**j**) and SEM (**d**, **f**) are indicated. Statistically significant differences were calculated by two-tailed Mann–Whitney test (*P < 0.05; **P < 0.01; ***P < 0.001, ****P < 0.0001).

The apoferritin nanocage scaffold is known to induce strong humoral immune responses^44,45^. To suppress the anti-particle response through glycan shielding, we engineered a modified immunogen (immunogen 145) by introducing two non-native glycosylation sites at position N79 and N99 of each apoferritin monomer. Expression of immunogen 145 in mammalian HEK293S cells [GnT I^−/−^] produced particles with homogeneous high mannose N-linked glycans at these positions (referred to as immunogen 145S, Fig. 3a). Similar to immunogen 126, 145S assembled into monodisperse, well-formed spherical particles (Fig. 3b, c), and bound strongly to mAbs reactive to the junction and NANP repeats, but not N- or C-CSP (Supplementary Fig. 4b). Immunization in wt mice with the glycosylated nanoparticle 145S adjuvanted with SAS lowered the anti-carrier response significantly compared to the parental immunogen 126 (Supplementary Fig. 7a, b), while the desired antibody response against the PfCSP epitopes in the junction and NANP repeats was strongly increased (Supplementary Fig. 7c, d). Similar results were observed with the adjuvant LMQ, a liposome-based adjuvant containing the saponin QS-21 and a synthetic TLR4 agonist^46^ resembling AS01 (Fig. 3d–g). Importantly, adjuvanting the glycan-engineered immunogen with LMQ induced high anti-PfCSP antibody responses after two immunizations (Supplementary Fig. 8).

The superior properties of the glycan-engineered immunogen 145S compared to the parental non-glycosylated immunogen 126 were also observed at the cellular level (Fig. 3h, i). Flow cytometric analyses showed significantly stronger anti-PfCSP B cell responses in draining lymph nodes after immunization with immunogen 145S compared to immunogen 126 (Fig. 3h, Supplementary Fig. 9). ELISpot analyses demonstrated that both immunogens induced the formation of PfCSP-reactive bone marrow (BM) plasma cells at high (10 µg) and at low (0.5 µg) immunization dose (Fig. 3i). Similar to the response in the periphery, the frequency of PfCSP-reactive BM plasma cells was significantly higher in mice immunized with immunogen 145S compared to immunogen 126, whereas *H. pylori* apoferritin-reactive BM plasma cells were much less abundant even after low-dose vaccination. Furthermore, sera from mice immunized with 145S had a higher capacity to inhibit Pf sporozoite traversal of hepatocytes in vitro than sera from mice immunized with immunogen 126 at both immunogen doses tested (Fig. 3j). We conclude that the engineering of N-linked glycosylation sites boosted the response against the protective PfCSP epitopes and efficiently shielded the nanocage carrier protein from the humoral immune response.

### Induction of strong T cell responses

**Fig. 4:**
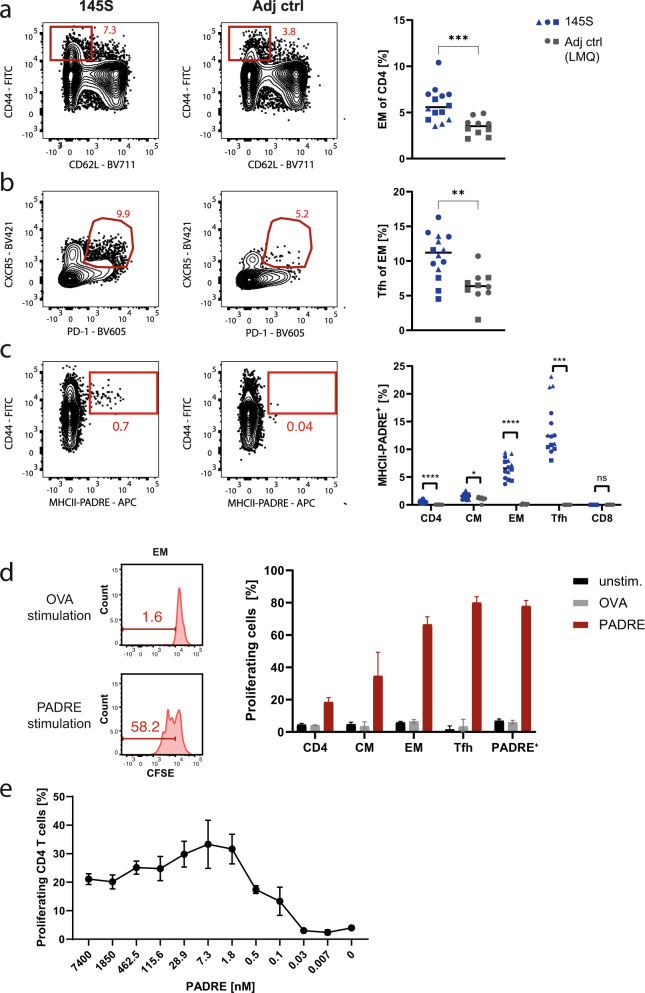
Efficient T cell help by the universal T cell epitope PADRE. C57BL/6J mice were immunized with immunogen 145S adjuvanted with LMQ at day 0 and 28 or adjuvant alone. Representative FACS plots (left) and quantification (right) of CD4 EM cells (**a**), EM Tfh cells (**b**) and PADRE-specific CD4 T cells (**c**) in lymph nodes 22 days after the last immunization. Detailed gating strategy is shown in Supplementary Fig. 10. Pooled data of three independent experiments for the immunogen 145S group and two independent experiments for the adjuvant control group with 5 mice per group are shown. Dots represent individual mice. Symbols indicate independent experiments. **d** In vitro CFSE proliferation assay with splenocytes isolated 22 days after the last immunization. Splenocytes were restimulated with 10 µg/ml (7.4 µM) PADRE or OVA control peptide and the proportion of CFSE^low^ cells was determined 3 days later. Representative FACS plots of EM T cells (left) after stimulation with the negative control OVA peptide (top) or PADRE (bottom). The proportion of proliferating cells was determined in different T cell populations including all gated PADRE+ CD4 T cells (right). Detailed gating strategy is shown in Supplementary Fig. 11. Pooled data of two independent experiments with two replicates for each experiment and 4 mice per group are shown. **e** The proportion of proliferating CD4 T cells 3 days after restimulation of splenocytes isolated 22 days after the last immunization with different concentrations of the PADRE peptide. Pooled data of two independent experiments with two replicates for each experiment and 4 mice per group are shown. Arithmetic mean (**a**–**e**) and SEM (**d**, **e**) are indicated. Statistically significant differences were calculated by two-tailed Mann–Whitney test (*P < 0.05; **P < 0.01; ***P < 0.001, ****P < 0.0001, n.s. statistically non-significant differences).

The induction of strong T helper cell responses is of great importance for the development of high-quality affinity matured and durable humoral immunity. To evaluate T cell responses induced by immunogen 145S, we determined the frequency of CD44^high^CD62L^neg^ effector memory (EM) and CXCR5^pos^PD-1^pos^ T follicular helper (Tfh) cells in mice immunized with immunogen 145S compared to adjuvant alone (Fig. 4a, b, Supplementary Fig. 10) and quantified the frequency of antigen-reactive T cells of different T cell populations using a PADRE-tetramer (Fig. 4c, Supplementary Fig. 10). Prime-boost immunization with immunogen 145S adjuvanted with LMQ induced strong EM and Tfh cells responses with high proportion of PADRE-reactive cells that mostly showed an activated (CD44^+^) phenotype (Fig. 4a–c). The frequency of antigen-specific cells was highest among Tfh cells (Fig. 4c) that also showed the strongest proliferative response upon PADRE restimulation ex vivo (Fig. 4d, Supplementary Fig. 11). The high potency of the PADRE T cell epitope was reflected by the extremely low peptide concentration of about 2 nM that was sufficient to induce proliferation (Fig. 4e). Of note, PADRE did not induce humoral responses much above background (Supplementary Fig. 12). Together, these data demonstrated the potency of the immunogen-encapsulated PADRE epitopes at inducing strong T helper cell responses and T cell memory.

### Long-lived protective immunity

**Fig. 5:**
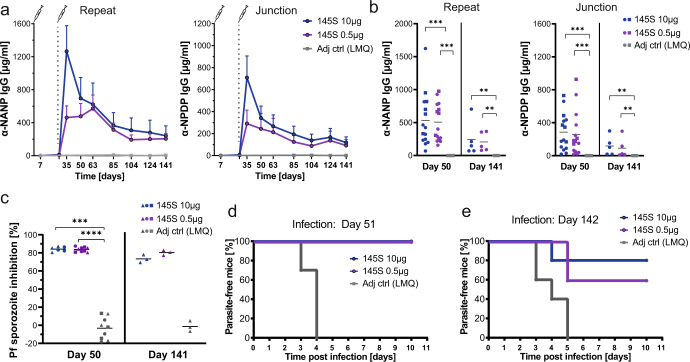
The glycosylated nanoparticle 145S confers long-lived protective antibody responses. **a** C57BL/6J mice were immunized with 10 µg or 0.5 µg immunogen 145S adjuvanted with LMQ or with LMQ alone at day 0 and 28. The serum IgG response against the repeat (NANP) and the junction (NPDP) was measured at different timepoints. One representative out of two independent experiments with 10 mice per group is shown until day 50. Day 51 until day 141 shows data from one experiment with 5 mice per group. **b** Anti-repeat (NANP) or junction (NPDP) IgG response at day 50 and day 141 after the first immunization. Dots represent individual mice. Day 50: Pooled data of three independent experiments with 5 mice per group are shown. Day 141: Data of one experiment with 5 mice per group is shown. Symbols indicate independent experiments. **c** Capacity of pooled sera (diluted 1:800) collected 50 days or 141 days after first immunization to inhibit the hepatocyte traversal activity of Pf sporozoites in vitro. Pooled data of three independent traversal assay experiments are shown. Symbols indicate independent immunization experiments. **d**, **e** Capacity of immunization with immunogen 145S at two different doses to protect mice after PbPfCSP(mCherry)-infected mosquito bite challenge (3 bites per mouse) from parasitemia. Data show the percentage of blood stage parasite-free mice over time. **d** Day of challenge 51: Pooled data of two independent experiments with 5 mice per group are shown. e Day of challenge 142: Data from one experiment with 5 mice per group. Arithmetic mean (**a**–**c**) and SEM (**a**) are indicated. Statistically-significant differences were calculated by two-tailed Mann–Whitney test (*P < 0.05; **P < 0.01; ***P < 0.001, ****P < 0.0001). Statistically non-significant differences are not indicated.

Next, we examined whether immunization with immunogen 145S would induce strong and durable antibody responses and protection against *Plasmodium* infection. Mice were immunized with formulations of 0.5 or 10 µg of immunogen 145S adjuvanted with LMQ following a prime-boost scheme. Robust antibody responses against the junction and NANP repeats were elicited at both doses (Fig. 5a). Although higher peak IgG levels were observed in the 10 µg group, the titers reached similar levels by day 50 after primary immunization and remained stable over 141 days (Fig. 5a, b). Independent of the immunogen dose, sera of the immunized mice efficiently blocked Pf sporozoite traversal of hepatocytes in vitro even 5 months after immunization (Fig. 5c). Immunized mice were exposed to the bites of mosquitoes infected with *P. berghei* transgenic mCherry reporter sporozoites expressing *PfCSP* instead of the endogenous *P. berghei* CSP, (PbPfCSP(mCherry) Supplementary Figs. 13, 14a–c) and the development of blood stage parasitaemia was measured by flow cytometry (Supplementary Fig. 14d). Challenge of the immunized mice under stringently controlled conditions with bites from three mosquitoes infected with PbPfCSP(mCherry) sporozoites 23 days after the booster immunization (day 51) showed sterile protection in all mice from the development of blood stage parasitemia (Fig. 5d). Importantly, a high level of protection was also observed when the immunized animals were challenged 5 months after immunization (day 142, Fig. 5e), where 4 mice and 3 mice out of 5 were protected after immunization with 10 and 0.5 ug, respectively. Thus, two immunizations of mice with immunogen 145S induced highly efficacious and durable immune responses against *Plasmodium* infections over a 20-fold dose range.

## Discussion

Here, we describe the design and engineering of a second-generation anti-infective malaria vaccine candidate following a reverse vaccinology 2.0^47^ approach guided by the molecular and cellular analysis of human anti-PfCSP antibody responses^10,24–32,34^. Our immunogens elicited strong, durable and protective humoral immunity against the most potent PfCSP epitopes in the junction and the repeat. The potency of antibodies against these target sites was associated with cross-reactivity to junction and repeat motifs^26^. Although B cells with NANP specificity dominated the immediate response to PfCSP on sporozoites in humans, cross-reactive antibodies were continuously selected due to their overall higher affinity^24,26^. GCs are essential mediators of affinity maturation and were efficiently induced by our immunogens, which were designed to improve the quality and the quantity of the humoral response. The high quality of the antibody response was also confirmed in the Kymouse™ model, where the selection of mutations known to increase the affinity of human anti-PfCSP antibodies and efficient class-switch recombination to IgG were clear indicators for efficient GC reactions and active affinity maturation.

The defining features of our immunogen design as compared to RTS,S and R21 – the inclusion of the junction, a low number of NANP motifs and the exclusion of the immunodominant C-CSP – might have helped focusing the response on the potent junction and repeat epitopes. Limiting the NANP repeat length and thereby valency likely increased the BCR activation threshold to selectively activate only precursors with strong antigen-binding capacity^30,34,40^. At the same time, it may have promoted the subsequent differentiation of these precursors into GC rather than antibody-secreting cells. The junction by itself seems to be poorly immunogenic^31^ but is often targeted by cross-reactive antibodies that recognize NANP motifs in their core epitope and gain junction affinity by somatic mutations^26^. Our results suggest that limiting the number of NANP motifs might be essential for the induction of strong anti-junction responses. Together, the data strongly support our strategy to focus the immune response on the target epitopes of the most potent human anti-PfCSP antibodies while avoiding the induction of non-inhibitory PfCSP antibodies, especially in the immunodominant C-terminus.

For efficient presentation to the immune system, we fused the PfCSP component to the N-terminus of *H. pylori* apoferritin, exposing the target B cell epitopes on the exterior of the nanoparticle. *H. pylori* apoferritin shows only 20% sequence similarity to human ferritin and no cross-reactivity has been reported during clinical evaluations^44,45^ (ClinicalTrials.gov, NCT03814720, NCT04579250). Immunogens that utilize the *H. pylori* apoferritin scaffold exhibit robust thermostability^48,49^ and high yields when adapted to a stable CHO manufacturing cell line^50^, illustrating the high translation potential of this scaffold. Presenting the PfCSP antigen on the surface of these nanoparticles drastically increased the anti-NANP serum response compared to monomeric PfCSP demonstrating that *H. pylori* apoferritin is a promising scaffold for the delivery of *Plasmodium* antigens.

The use of PADRE with its ability to elicit strong T cell responses via HLA-DR in humans (and via I-A^b^ in C57BL/6 mice)^51,52^ induced robust Ag-specific Tfh and potent memory responses linked to the generation of affinity matured long-lasting B cell immunity. Thus, PADRE compensated for the lack of the C-CSP, which contains the majority of all known T helper cell epitopes^13–18^. A potential drawback of choosing PADRE over PfCSP-derived epitopes could be that natural Pf infections will be unable to boost the vaccine-induced T cell memory response. However, the PfCSP C-terminus is highly polymorphic, and human T helper cells seem to be highly sensitive to the strong sequence variation in their target epitopes suggesting that boosting would be limited to infections with parasites that show high PfCSP sequence similarity to the vaccine strain, likely a rare natural event^13–18^.

The ability to hide T cell epitopes within the nanocage is an inherent advantage of the *H. pylori* apoferritin nanocage platform over other nanocage scaffolds, such as lumazine synthase or Tobacco mosaic virus (TMV)-like disks that allow physical access to peptide epitopes fused to the termini in the context of the assembled multimer. PADRE being a small peptide might not be expected to induce strong humoral responses; furthermore, as would be expected from its encapsulation inside the nanocage, we did not observe PADRE-specific antibodies to the fused T cell epitope after immunization. Future studies leveraging the *H. pylori* apoferritin nanocage platform might benefit from further exploration of size and immunogenicity considerations for concealed T cell epitopes.

The addition of non-native N-linked glycans onto the *H. pylori* apoferritin scaffold was another engineered feature that contributed to augmenting the quality of the immune response. In contrast to a recent study that investigated the effect of scaffold glycosylation in the context of viral glycoprotein antigens^53^, we observed that in addition to dampening of the anti-scaffold response, glycan modification of the *H. pylori* apoferritin nanocage boosted anti-PfCSP responses and increased the frequency of PfCSP-reactive B cells compared to the non-glycosylated immunogen. Both effects were also evident in the BM plasma cell compartment indicating a long-lasting effect and qualitative difference in the response. Previous studies have shown that mannosylated protein nanoparticles accumulated in lymph node follicles in a mannose-binding lectin (MBL) and complement dependent manner, and that this accumulation was associated with an enhanced humoral response^54,55^. Our findings demonstrate the potential benefits of including mannosylated glycans on nanoparticles to improve vaccine responses. In contrast to many viral glycoprotein antigens (such as HA in influenza, Env in HIV-1, S protein in SARS-CoV-2, or F protein in RSV), the PfCSP junction and NANP repeats lack N-linked glycosylation sites. Therefore, by introducing mannosylated N-linked glycans onto the scaffold of this Pf vaccine candidate, we likely utilized the MBL and complement pathway to improve the antigen-specific humoral response. Further studies will be required to dissect the mechanisms that decreased anti-carrier responses while boosting anti-PfCSP immunity by the engineered N-linked glycans and how different glycoforms of the apoferritin nanocage might impact these mechanisms.

The overall high protective capacity of our immunogen, even at the lowest tested dose and over the six months after immunization demonstrates the success of our reverse vaccinology-based approach. Strong support for its further development comes from the observation that our *H. pylori* apoferritin-scaffolded immunogen elicited VH3-33 antibodies with similar VK pairings and somatic hypermutation patterns as potent human mAbs^26,30,31,34^ in the context of the human variable gene repertoire of the Kymouse™ platform. Future preclinical and, ultimately, clinical studies will be required to address the optimal choice of adjuvant for this vaccine candidate and to evaluate the full extent of its efficacy and durability in humans.

## Methods

### Immunogen expression and purification

To express and purify FL-PfCSP (NF54, residues 20–384), FL-PfCSP was cloned into pHLsec for transient expression in HEK293F cells. Putative N-linked glycan sites encoded in the sequence were mutated to glutamine. FL-PfCSP was purified via HisTrap FF (Cytiva) and size exclusion chromatography (Superdex 200 Increase 10/300 GL, Cytiva), prior to immunization studies. To generate immunogen 126, a gene comprising a Strep-II tag, TEV cleavage site, the PfCSP junction epitope, NVDP and NANP motifs, a 15-aa linker, *H. pylori* apoferritin, a 3-aa linker and PADRE was synthesized by GeneArt and cloned into pHLsec for transient expression in HEK293F cells. Immunogen 126 was purified via StrepTrap HP (Cytiva) followed by TEV cleavage of the Strep-II tag for 3 h at room temperature (RT). Cleaved immunogen 126 was further purified via an additional StrepTrap HP (Cytiva) and size exclusion chromatography (Superose 6 Increase 10/300 GL, Cytiva) in PBS, prior to immunization studies. To generate immunogen 145 and introduce N-linked glycan sites onto the *H. pylori* apoferritin scaffold at positions 79 and 99, we introduced the following mutations; K79N, E81T, E99N and I101T. As recently reported, some nanoparticles with a high mannose content tended to exhibit more efficient follicular localization and stronger antibody responses compared to those with native, complex glycans^54^. Therefore, immunogen 145 was transiently transfected in HEK293S (GnT I^−/−^) cells to generate immunogen 145S.

### Negative stain electron microscopy

3 μL of immunogens 126 and 145S were applied to glow-discharged carbon grids. After 20 s, the grid was blotted and 3 μL of 2 % (w/v) uranyl formate solution was applied to the grid three times for two lots of 5 s and a final 18 s, with blots in between. Data were collected on a FEI Tecnai 20 operated at 200 kV.

### Biolayer interferometry binding studies

BLI (Octet RED96, Sartorius) experiments were conducted to test whether immunogens could be recognized by mAbs reactive to the junction and NANP motifs. mAbs CIS43, 1210, 1710, and 5D5 were diluted to 10 μg/mL in kinetics buffer (PBS, pH 7.4, 0.1 % (w/v) BSA, and 0.02% Tween20) and immobilized onto FAB2G biosensors. Following the establishment of a stable baseline with loaded ligand in kinetics buffer, biosensors were dipped into wells containing immunogen 126 or 145S. Tips were then dipped back into kinetics buffer to monitor the dissociation rate. Analysis of affinity constants was not attempted, as the high avidity of the interactions resulted in negligible dissociation rates.

### LMQ preparation, formulations with immunogens and characterization

LMQ adjuvant was developed by and manufactured at the Vaccine Formulation Institute by mixing a solution of QS21 (Desert King International, CA, USA) in phosphate buffered saline (PBS) with liposomes containing the synthetic TLR4 agonist 3D-(6-acyl) PHAD (3D6AP; Merck-Avanti 699855P, USA). Liposomes were prepared by the lipid film method, using 1,2-dioleoyl-sn-glycero-3-phosphocholine (DOPC) and cholesterol as lipids. The TLR4 agonist 3D6AP was incorporated within the lipid film. Rehydration of the lipid film was done in PBS followed by extrusion to yield liposomes. To evaluate immunogens compatibility with adjuvant, stability of formulations containing the immunogens in combination with LMQ was monitored after storage for 24 h at 5 °C. Formulations were characterized by visual inspection, particle size, polydispersity, zeta potential, pH and osmolality. The lipid content was also measured by High Performance Liquid Chromatography-UV (Agilent Technologies, CA, US) and QS21/3D6AP amounts measured by Ultra Performance Liquid Chromatography-Mass Spectrometry (Waters, MA, US).

### Mice

For immunization experiments, female C57BL/6J mice (Janvier labs) were maintained at a specific pathogen-free facility of the Central Animal Laboratory of the German Cancer Research Center. All C57BL/6J mouse procedures were approved by the regional authorities in Karlsruhe, Germany (project numbers G-255/17 and G-278/20) and experiments were conducted in accordance with the German Animal Protection Law.

Male and female transgenic mice from the Kymouse™ platform^42^ were housed and all procedures carried out under United Kingdom Home Office License 70/8718 with the approval of the Wellcome Trust Sanger Institute Animal Welfare and Ethical Review Body.

For generation of the new PbPfCSP(mCherry) reporter line, female OF1 mice (6–7-week old; Charles River, NL) were used. All animal experiments were granted with a license by Competent Authority after an advice on the ethical evaluation by the Animal Experiments Committee Leiden (AVD1160020171625). All experiments were performed in accordance with the Experiments on Animals Act (Wod, 2014), the applicable legislation in the Netherlands in accordance with the European guidelines (EU directive no. 2010/63/EU) regarding the protection of animals used for scientific purposes. All experiments were executed in a licensed establishment for the use of experimental animals (LUMC). Mice were housed in individually ventilated cages furnished with autoclaved aspen woodchip, fun tunnel, wood chew block and nestlets at 21 ± 2 °C under a 12:12 h light-dark cycle at a relative humidity of 55 ± 10%.

For mosquito infections with PbPfCSP(mCherry) parasites, female CD1 mice (7–12-week old) and female C57BL/6J mice (14-week old) were bred in the MPIIB Experimental Animal Facility (Marienfelde, Berlin) and housed in a pathogen-free animal facility at the Max Planck Institute for Infection Biology in individually ventilated cages containing nestlets, fun tunnel and wood chew block. Mice were handled in accordance with the German Animal Protection Law (§8 Tierschutzgesetz) and approved by the Landesamt für Gesundheit und Soziales (LAGeSo), Berlin, Germany (project numbers 368/12 and H0335/17).

### Mouse immunizations

Immunogens were mixed 1:1 with Sigma Adjuvant System (SAS, Sigma) according to manufacturer’s instruction or with LMQ (as described above). Adjuvant control animals received a PBS-adjuvant mixture. Mice were immunized at an age of 7–9 weeks, according to the immunization schedule and immunogen dose mentioned in the figure legends. Each mouse received 100 μl immunogen-SAS mixture subcutaneously (s.c.) at the right and left side of the tail base or 25 μl immunogen-LMQ mixture injected intramuscularly (i.m.) into the right and left thigh muscle.

### Blood and organ collections

Peripheral whole blood was collected from the submandibular vein, allowed to clot for 1–2 h at RT and kept at 4 °C for 4–16 h. Serum was collected after spinning at 2350 g in a top-bench centrifuge for 5 min at 4 °C, and stored at −20 °C.

Mice were sacrificed 7 days after the final boost by cervical dislocation (C57BL/6J mice) or under UK Home Office Schedule 1 by rising concentration of CO_2_ (mice from the Kymouse™ platform). Popliteal, inguinal and axial lymph nodes, spleens, and bone marrow were collected. Single-cell suspensions were prepared by mashing cells through a 40 μm cell strainer (BD).

### Enzyme-linked immunosorbent assay (ELISA)

Antigen ELISAs were performed as described^27^. In brief, high-binding 384-well polystyrene plates (Corning) were coated overnight at 4 °C with 2 µg/ml NPDPNANPNVDPNANP (junction,) (NANP)_5_ (repeat), SLGENDDGNNEDNEKLRKPKHKKLKQPADGNPDP (N-CSP), NKNNQGNGQGHNMPNDPNRNVDENANANSAVKNNNNEEPSDKHIKEYLNKIQNSLSTEWSPCSVTCGNGIQVRIKPGSANKPKDELDYANDIEKKICKMEKCSSVFNVVNSS (C-CSP), 0.7 µg/ml AKFVAAWTLKAAA (PADRE) or 1 µg/ml *H. pylori* apoferritin in 20 µl. Plates were washed three times with 0.05% Tween 20 in PBS, blocked with 50 µL of 4% BSA in PBS for 1 h at RT, and washed again prior to incubation with 20µL per well of serum samples diluted in 1% BSA in PBS for 90 min at RT. Wells were washed six times and incubated with goat anti-mouse IgG-HRP at 1:1000 (Jackson Immuno Research) in PBS with 1% BSA for 1 h. Wells were washed again and one-step ABTS substrate (RT, 20 µL per well; Roche) and 1× KPL ABTS peroxidase stop solution (RT, 20 µL per well; SeraCare Life Sciences) were used for detection. The concentration of antigen-specific IgG was determined by comparison to an IgG1 standard curve (BD Pharming) of known concentration on each plate.

### Enzyme-linked immunospot assay (ELISpot)

The ELISpot assay was conducted according to the manufacturer´s protocol (Mouse IgG/IgM double color ELISpot kit, Immunospot). Briefly, 96-well assay plates were coated overnight with 1 µg/ml FL-PfCSP (3D7) or *H. pylori* apoferritin. The next day, primary bone marrow cells were seeded at a density of 0.5 × 10^6^ cells/well for each antigen. Plates were incubated for 16–18 h in a cell culture incubator (37 °C, 8% CO_2_) before being developed. Spots were automatically counted using a CTL immunospot reader.

### Serum NANP antibody depletion

HisPur Cobalt Resin (50 µl, Thermo Fisher) was washed with binding buffer (300 mM NaCl and 50 mM Na_3_PO_4_) twice by spinning at 700 × g for 2 min followed by removal of the supernatant and coated with 80 µg/ml (NANP)_10_H_6_ in binding buffer for 3 h at 4 °C rotating. Beads were washed again and blocked for 3 h with 4% BSA in PBS at RT rotating. Sera of mice from each group were pooled and 200 µl of pooled sera was diluted 1:1 in imidazole buffer (20 mM imidazole, 300 mM NaCl, 50 mM Na_3_PO_4_) were added to the beads that had been washed again and incubated for a minimum of 2 h at 4 °C rotating. Beads were spun down and supernatant was transferred to a new tube with coated and washed beads. This procedure was repeated two times before further analysis in ELISA or Pf sporozoite hepatocyte traversal assay.

### CFSE proliferation assay

Splenocytes (1 × 10^7^) were resuspended in 1 ml PBS. 1 ml of 1 µM CFSE (Sigma) solution in PBS was added slowly to the tube followed by incubation for 8–10 min at 37 °C. The reaction was stopped by adding pre-warmed 10% FCS in PBS solution. Cells were washed in PBS and resuspended in 1 ml DMEM containing 10% FCS, 0.01 M Hepes, 2 mM L-Glutamine, 0.05 mM β-Mercaptoethanol, 100 IU/mL penicillin, 100 µg/mL streptomycin. Cells were plated at a concentration of 5 × 10^5^ cells/200 µL to a 96-well round U bottom plate without or with PADRE or OVALBUMIN at various concentrations mentioned in the figure legends and incubated for three days at 37 °C and 8% CO_2_.

### Preparation of p126-SA tetramers for flow cytometric analysis

Peptide p126 (GKQPADGNPDPNANPNVDPNANPNVDPNANPNVDPNANPNANPNANPNANPNANP, Peptide Specialty Laboratories GmbH, Heidelberg) at a concentration of 0.64 μg/µl in PBS was slowly mixed with either SA-PE (Agilent Technologies) or SA-APC (Agilent Technologies) in a 1:4.1 molar ratio. After overnight incubation at 4 °C in the dark, sterile PBS was added before transfer to a 50K membrane filtration tube (Merck-Millipore) and centrifugation for 5 min at 8,000 x g until about 50 μl were left. After washing the tetramers once by repeating the above procedure, filters were inserted upside down into a collection tube and centrifuged for 3 min at 1,000 x g. Subsequently, 100 μl of sterile PBS was added to the filter and centrifuged for collection into the same tube as described above. After repeating the previous step, both tetramers were mixed and topped up to a concentration of 87.5 pmol/ml with PBS containing 2% FCS to be used in FACS.

### Flow cytometric analysis

Single-cell suspensions from lymph nodes, bone marrow or spleen cell cultures (CFSE proliferation assay) of C57BL/6J mice were incubated for 15 min at 4 °C with anti-CD16/CD32 Fc-receptor block (2.4G2, eBiosciences, #14-0161-82, 1:100), followed by incubation for 1 h at RT with p126-SA tetramers or PADRE-MHCII tetramers (I-A(b) synthetic epitope PADRE AKFVAAWTLKAA APC-Labeled Tetramer (NIH)). Cells were then washed and stained with anti-CD4-APC-Cy7 (GK1.5, Biolegend, #100414, 1:100), anti-CD8-APC-R700 (53–6.7, BD Biosciences, #564983, 1:200), anti-CD44- BV785 (IM7, Biolegend, #103041, 1:100), anti-CD44- FITC (IM7, BD Pharmigen, #553133), anti-CD62L-BV711 (MEL-14, Biolegend, #104445, 1:800), anti-PD-1-BV605 (J43, BD Biosciences, #563059, 1:100), anti-CXCR5-BV421 (L138D7, Biolegend, #145511, 1:400), anti-GL-7-FITC (GL7, BD Pharmigen, #562080, 1:1000), anti-CD38-Pe-Cy7 (90, Biolegend, #102718, 1:400), anti-IgD-APC-Cy7 (11-26c.2a, Biolegend, #405715, 1:1000), anti-CD19-APC-Red (1D3, BD Biosciences, #565473, 1:400), anti-IgM-BV786 (II/41, BD Biosciences, #743328, 1:100), anti-IgG1-BV510 (RMG1-1, Biolegend, #406621, 1:100), anti-IgG2a/b-BV510 (R2-40, BD Biosciences, #744293, 1:100), anti-IgG3-BV510 (R40-82, BD Biosciences, #744134, 1:100) and anti-TACI-BV421 (8F10, BD Biosciences, #742840, 1:200) antibody respectively, all diluted in PBS containing 2% FCS. Dead cells were excluded using 7-Aminoactinomycin D (7AAD, Life Technologies).

Single-cell preparation from lymph nodes of mice of the Kymouse™ platform were incubated for 10 min at 4 °C with TruStain FcX/Fc blocker (Biolegend), followed by incubation for 30 min at 4 °C with FL-PfCSP-SA-APC tetramers, generated by incubation in a (4:1 CSP: Streptavidin (Prozyme) ratio) for 30 min at 4 °C and with anti-CD19-BV510 (6D5, Biolegend, #115546, 1:80), anti-CD45R/B220-BUV395 (RA3-6B2, BD Biosciences, #563793, 1:80), anti-GL7-eFluor450 (GL7, eBiosience, #48-5902-82, 1:80), anti-CD95-PE-Cy7 (Jo2, BD Biosciences, #557653, 1:80) all diluted in Brilliant stain buffer (BD Biosciences). Dead cells were excluded using Draq7 (BioStatus) together with the following antibodies staining Dump channel positive cells: anti-CD8-APC-Cy7 (53–6.7, BD Bioscience, #557654, 1:80), anti-CD4-APC-Cy7 (GK1.5, BD Bioscience, #552051, 1:80), anti-Ly-6G-APC-Cy7(RB6-8C5, BD Pharmingen, #557661, 1:80), anti-F4/80-APC-Cy7 (BM8, Biolegend, #123118, 1:80) and anti-CD11c-APC-Cy7 (HL3, BD Pharmingen, #561241, 1:80). Data were collected on a BD FACSAria^TM^ III cell sorter, using the BD FACSDiva^TM^ software. Cell events were analyzed using the FlowJo v10.7.2 software.

### Mosquitoes

All mosquitoes were kept at 28–30 °C, 70–80% humidity and 12:12 h light-dark cycle. *Anopheles coluzzii* Ngousso *S1* strain^56^ was used for the production of Pf NF54 sporozoites for in vitro traversal assays and sporozoite binding assays. *A. gambiae 7b* line, immunocompromised transgenic mosquitoes derived from the G3 laboratory strain^57^, were used for the production of PbPfCSP(mCherry) sporozoites for in vivo infections.

### *P. falciparum* cultures

*P. falciparum* NF54 parasites (a kind gift of R. Sauerwein) were cultured in O+ human red blood cells (Haema, Berlin) at 37 °C, 4% CO_2_ and 3% O_2_ in a Heracell 150i Tri-gas incubator (Thermo Fisher Scientific). For gametocyte production, asynchronous asexual parasite cultures were diluted to 1% parasitemia and 4% hematocrit and maintained for 15–16 d with daily change of RPMI-1640 medium (Thermo Fisher Scientific) supplemented with 10% human A+ serum (Haema, Berlin) and 10 mM hypoxanthine (c-c-Pro) until mosquito infections.

### *P. falciparum* sporozoite hepatocyte traversal assay

The human hepatocyte line HC-04^58^ was cultured at 37 °C and 5% CO_2_ in HC-04 medium (MEM (- L-glu, Gibco) and F-12 Nutrient Mix (+L-glu, Gibco) 1:1 vol/vol mix, 15 mM HEPES, 1.5 g/l NaHCO_3_, 2.5 mM additional L-glutamine, 10% FCS and 1X Penicillin-Streptomycin (Gibco)).

*A. coluzzii* mosquitoes were infected with mature Pf gametocytes via artificial midi-feeders (Glass Instruments, the Netherlands) for 15 min and kept at 26 °C and 80% humidity in a controlled S3 facility in accordance with local safety authorizations (Landesamt für Gesundheit und Soziales Berlin, Germany, LAGeSo, project number 297/13). Infected mosquitoes received an additional uninfected blood meal 8 days post-infection (dpi) and were collected at 13–15 dpi. Sporozoites were isolated in HC-04 medium by dissecting and grinding mosquito thorax portions containing the salivary glands with glass pestles, followed by filtering the extracts with a 40 µm cell strainer (BD Biosciences). The isolated salivary gland sporozoites were enumerated in a hemocytometer (Malassez) and used for traversal assays. Sera from individual mice from the same experimental group were pooled in an equivolumetric fashion. Salivary gland Pf sporozoites in HC-04 medium were pre-incubated with diluted pooled serum samples for 30 min on ice and added to HC-04 cells for 2 h at 37 °C and 5% CO_2_ in the presence of 0.5 mg mL^–1^ dextran-rhodamine (Molecular Probes). Cells were washed, trypsinized and fixed with 1% PFA in PBS before measuring dextran positivity using a FACS LSR II instrument (BD Biosciences). Data analysis was performed by subtraction of the background (dextran positivity in cells treated with uninfected mosquito salivary gland material) and normalization to the maximum Pf traversal capacity (dextran positivity in cells treated with salivary gland Pf sporozoites without serum) using FlowJo V.10.0.8 (Tree Star) (Supplementary Fig. 2).

### *Transgenic P. berghei* parasite lines expressing PfCSP

To generate transgenic *P. berghei* parasites expressing PfCSP and the reporter mCherry (PbPfCSP(mCherry)), the published reference reporter *P. berghei* (ANKA) line expressing mCherry and luciferase under the constitutive *hsp70* and *eef1a* promotors, respectively (*Pb*-mCherry_hsp70_-Luc_eef1a_; line 1868cl1, RMgm-1320, www.pberghei.eu) was used^59^. To generate a chimeric parasite line that has the *Pbcsp* gene (PBANKA_0403200) replaced with the *Pfcsp* gene (PF3D7_0304600/PfNF54_030009700) we used a 2-step gene insertion/marker out transfection protocol (GIMO)^60^. In the first step, we deleted the *Pbcsp* coding open reading frame (ORF) and replaced it with the positive-negative selectable marker (SM), to create a *P. berghei CSP* deletion GIMO line (*PbCSP* GIMO). For this purpose, we generated the *pL2153* construct that is based on the standard GIMO DNA construct *pL0034* (MRA-849, www.beiresources.org). This construct contains the positive-negative (*hdhfr::yfcu*) SM cassette, and was used to insert both the *PbCSP* 5’- and 3’-gene targeting regions (TR), encompassing the full-length promoter and transcription terminator (TR) sequences, respectively. The construct was linearized using *Apa* I*, Xho* I and *Sca* I restriction sites outside of the 5’- and 3’-TRs before transfection. *Pb*-mCherry_hsp70_-Luc_eef1a_ parasites were transfected with *pL2153* using standard methods of GIMO-transfection^60^. Transfected parasites were selected by positive selection in mice by providing pyrimethamine in the drinking water^61^. Cloning of selected parasites resulted in line 3065cl1 *Pb*-mCherry_hsp70_-Luc_eef1a_-*CSP*-GIMO. Correct integration of DNA construct into the genome of parasites of line 3065cl1 was verified by Southern analyses of Pulsed Field Gel (PFG)-separated chromosomes and by diagnostic PCR analysis^60^. PFG-separated chromosomes were hybridized with a mixture of two probes: a probe recognizing the *hdhfr* gene and a control probe recognizing gene PBANKA_0508000 on chromosome 5^62^. PCR primers used to confirm correct integration of the construct into the *CSP* locus are shown in Supplementary Fig. 13. Primer sequences are listed in Supplementary Table 1.

In the second step, the positive-negative SM in the *Pb*-mCherry_hsp70_-Luc_eef1a_-*CSP*-GIMO parasites (line 3065cl1) was replaced with the *Pfcsp* gene by GIMO transfection, using construct *pL1972* containing *Pfcsp* (PF3D7_0304600/PfNF54_030009700) flanked by the *Pbcsp* 5’- and 3’-gene targeting regions^27^. The construct was linearized using *Afl* II and *Sac* I restriction sites before transfection and used to transfect parasites using standard methods of GIMO-transfection^60^. Transfected parasites were selected in mice by applying negative selection by providing 5-fluorocytosine (5-FC) in the drinking water^60^. Transgenic parasites with the *hdhfr::yfcu* SM in the *csp* locus of *Pb*-mCherry_hsp70_-Luc_eef1a_-*CSP*-GIMO parasites replaced by the *Pfcsp* gene, were selected by negative selection with 5-FC and cloned by limiting dilution resulting in PbPfCSP(mCherry) parasites (line 3079cl1). Correct integration of DNA construct into the genome was verified by Southern analyses of Pulsed Field Gel (PFG)-separated chromosomes and by diagnostic PCR analysis^60^. PFG-separated chromosomes were hybridized with a mixture of two probes: a probe recognizing the *hdhfr* gene and a control probe recognizing gene fragment of PBANKA_0508000 on chromosome 5^62^. PCR primers used to confirm correct integration of the construct into the *PfCSP* locus are shown in Supplementary Fig. 13. Primer sequences are listed in Supplementary Table 1.

### Challenge of immunized mice with PbPfCSP(mCherry) sporozoites

PbPfCSP(mCherry) blood stage parasites were passaged every 3–4 d in CD1 female mice, and *A. gambiae 7b* mosquitoes were infected (0.5–0.9% gametocytemia), kept at 20 °C and 80% humidity and offered an additional uninfected blood meal at 7 dpi. PbPfCSP(mCherry)-infected mosquitoes were knock down on ice at 17 dpi and selected for mCherry signal in the salivary glands under a fluorescence stereo microscope (Leica, M205 FA, Supplementary Fig. 14a). Salivary gland-positive mosquitoes were singled out in individual containers and starved overnight. The next day (18 dpi), anesthetized naïve C57BL/6J mice (14-week old) were exposed for 10 min to three or five bites of the presorted PbPfCSP(mCherry)-infected mosquitoes. Mouse blood was collected from the tail vein in heparinized capillaries (Brand) in PBS and parasitemia (mCherry-positive red blood cells / total red blood cells) was measured from days 3 to 7 and on day 10 post mosquito bite by flow cytometry (LSR II instrument, BD Biosciences). Blood stage positivity and negativity were confirmed by Giemsa-stained thin blood smears before mouse euthanasia and on day 10 post mosquito bite, respectively. All infected mice were euthanized on day 7 post mosquito bite, before the occurrence of malaria symptoms. FACS data were analyzed by FlowJo V.10.0.8 and the prepatency period was declared on the first day when parasitaemia values were above the background signal from negative mice. In these controlled conditions, three bites from salivary gland-positive mosquitoes were sufficient for 100% patent infection in naïve mice at day 5 post mosquito bite (*n* = 10, Supplementary Fig. 14b). Therefore, 3 bites were chosen as a controlled inoculum dose in the immunization experiments. Challenge by mosquito bite was performed in immunized mice (51 or 141 days post immunization) as detailed above (Supplementary Fig. 14c).

### Quantification of serum-antibody binding to live Pf sporozoites by flow cytometry

Pf sporozoites were isolated from mosquito salivary glands 13–15 dpi using the same procedures described for traversal assay. Siliconized microcentrifuge tubes (Alpha Laboratories) and pipet tips (VWR) were used to minimize sporozoite binding to the surfaces. Immunized mice pooled sera pre or post NANP antibody depletion (dilution 1:100) were incubated with 150,000 sporozoites in a total volume of 100 µl PBS with 1% FCS for 30 min at 4 °C. Upon washing, the sporozoites were incubated with Alexa Fluor® 555 Goat Anti-Mouse IgG (Molecular Probes; A-21422, 1:1,000) and SYBR Green I nuclear dye (Thermo Scientific; S7563, 1:10,000) in PBS with 1% BSA for 30 min at 4 °C. After washing, the live sporozoites were identified by SYBR Green signal and serum-antibody binding was quantified using FACS LSR II instrument (BD Biosciences). Data analysis was performed using FlowJo V.10.0.8 (Tree Star).

### Single-cell analysis

Spleens, lymph nodes and bone marrow isolated from each mouse from the Kymouse™ platform were processed to single-cell suspensions, cryopreserved in 10% DMSO/FBS and stored in liquid nitrogen. The target population, CD19^+^ B220^+^ GL7^+^ CD95^+^ germinal center B cells from spleen and lymph nodes was sorted into individual wells in a 96-well plate filled with lysis buffer (RNA Lysis buffer from QuickExtract™ RNA Extraction Kit, Epicentre, QER090150) using the BD FACS Aria Fusion flow cytometer (Beckton Dickinson). RT-PCR was performed to amplify the VH and VL domains, and standard Illumina libraries were generated before sequencing on an Illumina MiSeq. Reads corresponding to the same plate/well location were combined into consensus sequences. Germline assignment and sequence annotation of the consensus sequences was performed using the Kymab seq-utils Java program that automates the constant-region binning and identifies the V(D)J segments in large sets of antibody transcript sequences^42^. Sequence analysis was performed using R version 4.2.2^63^.

### Statistics

Statistical analysis was performed using Prism 7.04 (GraphPad) or R version 4.2.2^63^ using two-tailed Mann–Whitney assuming non-normal distribution, Kruskal–Wallis test with Dunn’s correction or two-tailed Mantel–Cox log-rank test for in vivo experiments, as described in the figure legends. *P* values below 0.05 were considered significant and indicated by asterisks.

### Reporting summary

Further information on research design is available in the Nature Research Reporting Summary linked to this article.

## Supplementary information

Supplemental Material

REPORTING SUMMARY

## Data Availability

All data generated or analyzed during this study are included in this published article (and its supplementary information files). The data supporting this study’s findings are available from the corresponding authors upon reasonable request.
