# Author Correction: Glycosylated nanoparticle-based PfCSP vaccine confers long-lasting antibody responses and sterile protection in mouse malaria model

**DOI:** 10.1038/s41541-023-00687-x

**Published:** 2023-06-05

**Authors:** Julia Ludwig, Stephen W. Scally, Giulia Costa, Sandro Hoffmann, Rajagopal Murugan, Jana Lossin, Katherine Prieto, Anna Obraztsova, Nina Lobeto, Blandine Franke-Fayard, Chris J. Janse, Celia Lebas, Nicolas Collin, Spela Binter, Paul Kellam, Elena A. Levashina, Hedda Wardemann, Jean-Philippe Julien

**Affiliations:** 1 B Cell Immunology, German Cancer Research Center (DKFZ), Heidelberg, Germany; 2 Program in Molecular Medicine, The Hospital for Sick Children Research Institute, Toronto, ON Canada; 3 Department of Immunology, University of Toronto, Toronto, ON Canada; 4 Vector Biology Unit, Max Planck Institute for Infection Biology, Berlin, Germany; 5 Malaria Research Group, Department of Parasitology, Leiden University Medical Center, Leiden, The Netherlands; 6 Vaccine Formulation Institute, Plan-les-Ouates, Switzerland; 7 Kymab a Sanofi Company, Babraham Research Campus, Cambridge, UK; 8 Department of Infectious Diseases, Faculty of Medicine, Imperial College London, London, UK; 9 Department of Biochemistry, University of Toronto, Toronto, ON Canada

**Keywords:** Malaria, Protein vaccines

Correction to: *npj Vaccines* 10.1038/s41541-023-00653-7, published online 07 April 2023

In this article the author name Anna Obraztsova was incorrectly written as Anna Obraztcova. The original article has been corrected.

