# Supplementary material for: Glycosylated nanoparticle-based PfCSP vaccine confers long-lasting antibody responses and sterile protection in mouse malaria model
Source: NPJ Vaccines. 2023 Apr 7;8:52. doi: 10.1038/s41541-023-00653-7 (PMC10080175; doi:10.1038/s41541-023-00653-7)
Supplement: Supplementary file 1 — Supplemental Material [file 41541_2023_653_MOESM1_ESM.pdf]

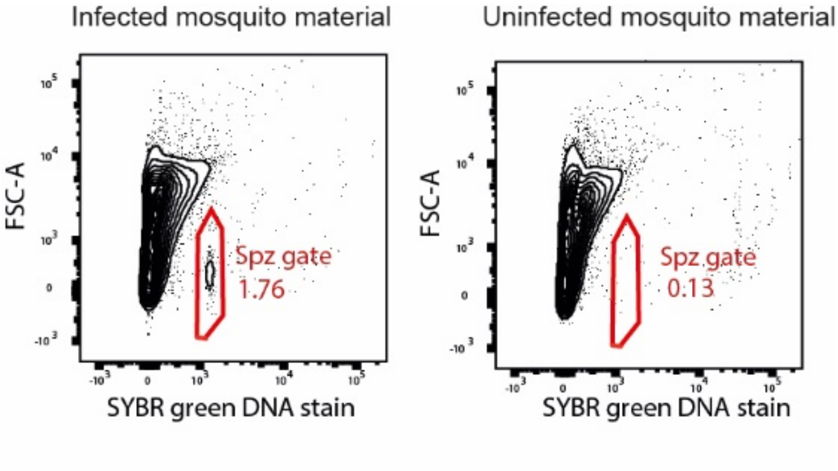

Suppl. Fig. 1 Gating of Pf sporozoites from salivary glands (Fig. 1c)

Representative flow cytometric gating strategy for the detection of Pf salivary gland sporozoites (spz) with SYBR Green nuclear staining. Salivary gland material from uninfected mosquitoes was used as gating control.

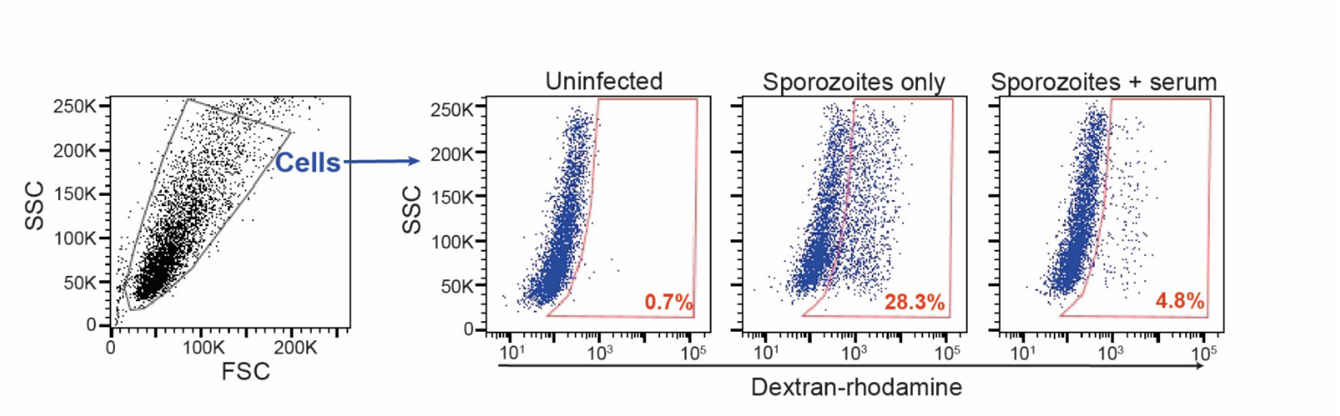

Suppl. Fig. 2 Gating strategy for Pf traversal assay (Fig. 1c)

Representative flow cytometric gating strategy for Pf sporozoite traversal assay. HC-04 cells are gated on FSC and SSC parameters (cells), and traversed cells are detected as Dextran-rhodamine positive (red gate). Background fluorescence in presence of uninfected salivary gland mosquito material (uninfected) was subtracted.

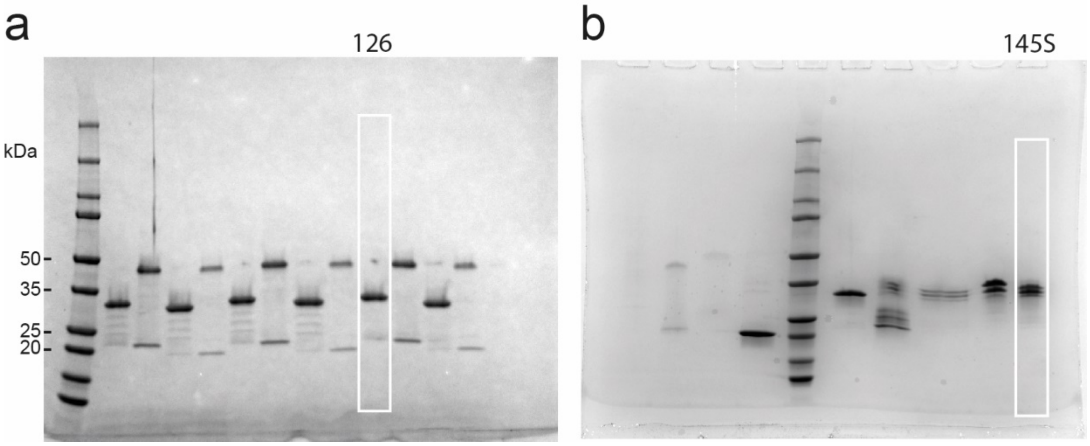

Suppl. Fig. 3 Uncropped SDS-PAGE analysis of immunogens 126 and 145S

Representative SDS-PAGE analysis of immunogens 126 (a) and 145S (b).

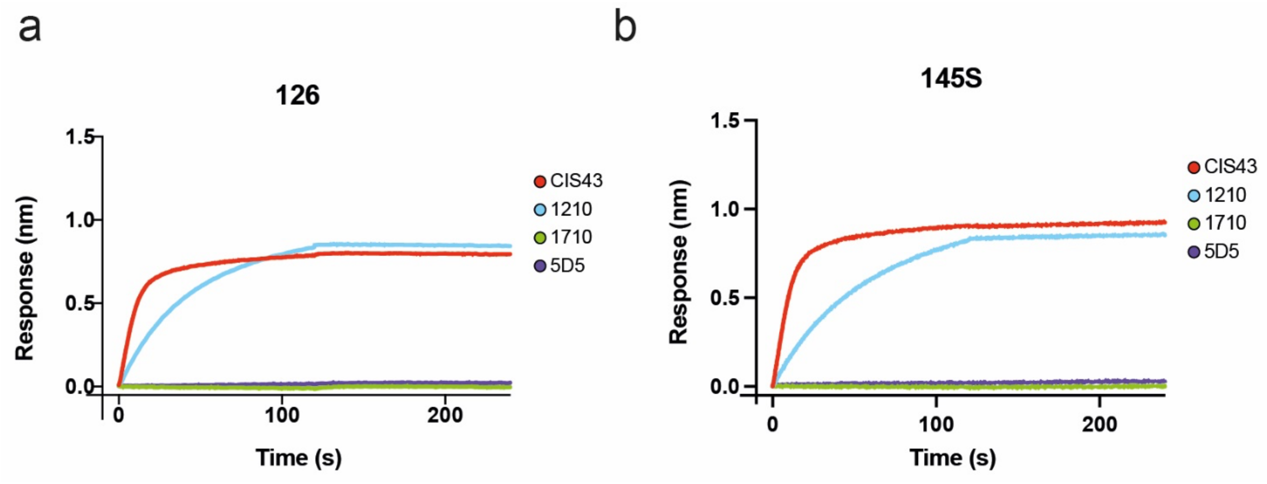

Suppl. Fig. 4 Biolayer interferometry binding studies

Representative sensorgrams of antibody binding to immunogens, including anti-junction antibody CIS43, anti-NANP antibody 1210, anti-C-CSP antibody 1710 and anti-N-CSP antibody 5D5 binding to (a) immunogen 126 and (b) immunogen 145S.

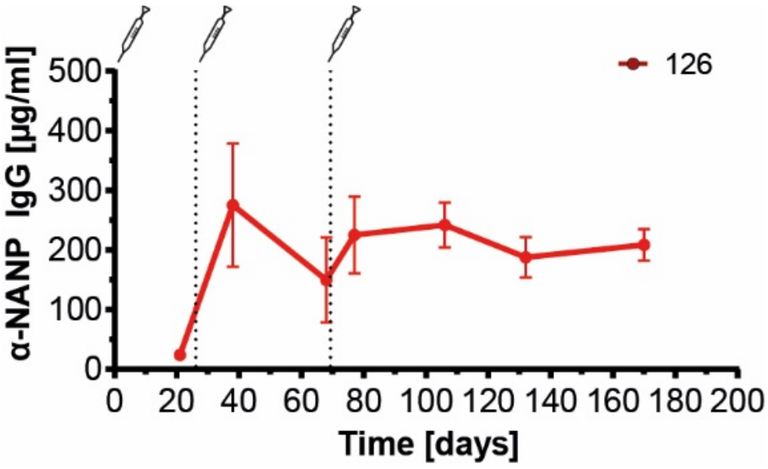

Suppl. Fig. 5 Long term response of immunization with immunogen 126 adjuvanted with SAS

C57BL/6/J mice were immunized with immunogen 126 adjuvanted with SAS at day 0, 28 and 42. The serum IgG response against the PfCSP repeat (NANP) was measured at various timepoints. Data of one experiment with 5 mice per group are shown. Arithmetic mean and SEM is indicated.

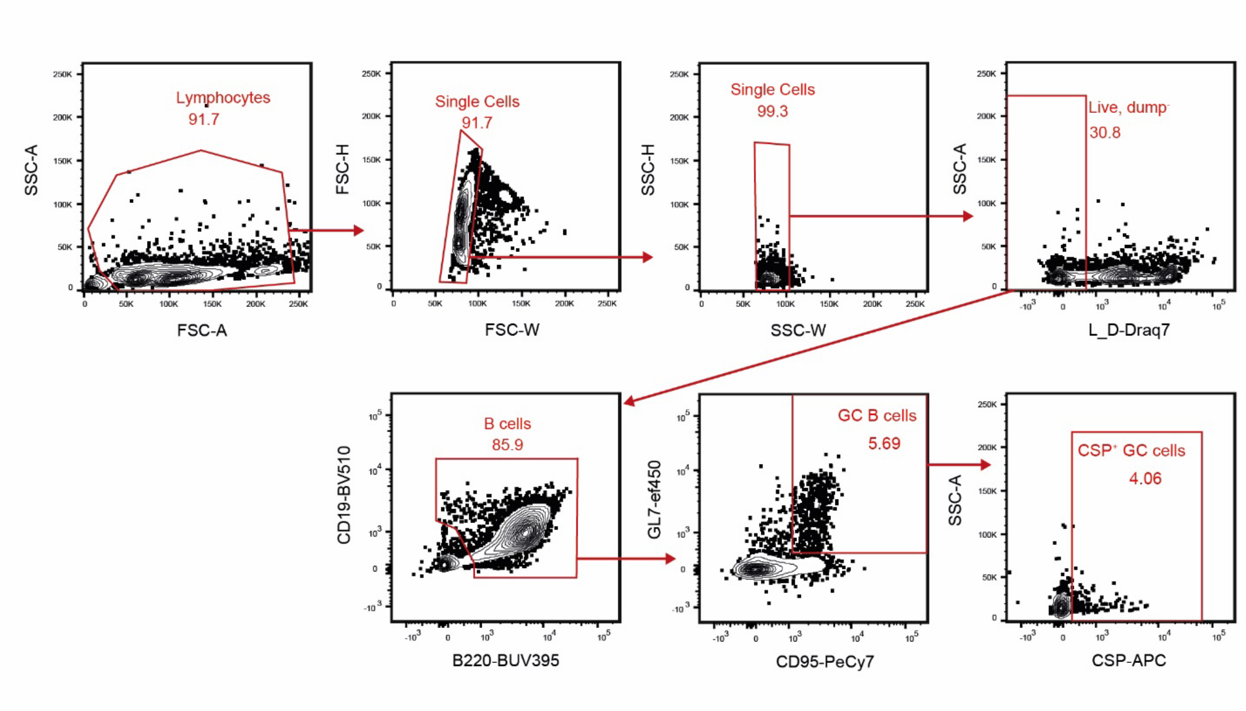

Suppl. Fig. 6 Gating strategy to detect CSP$^+$ Germinal center (GC) B cells for single-cell analysis

Representative flow cytometric gating strategy for the detection of CSP$^+$ GC B cells in mice from the Kymouse™ platform. Lymphocytes are gated by Forward and Sideward Scatter parameters (FSC-A and SSC-A), duplicates are excluded by size parameters (FSC-H and FSC-W) and Sideward Scatter parameters (SSC-H and SSC-W) and live and dump^{neg} cells are used for further analysis. GL7$^+$CD95$^+$ GC B cells are gated from CD19$^+$B220$^+$ B cells. CSP$^+$ were considered antigen specific and were sorted for single cell analysis.

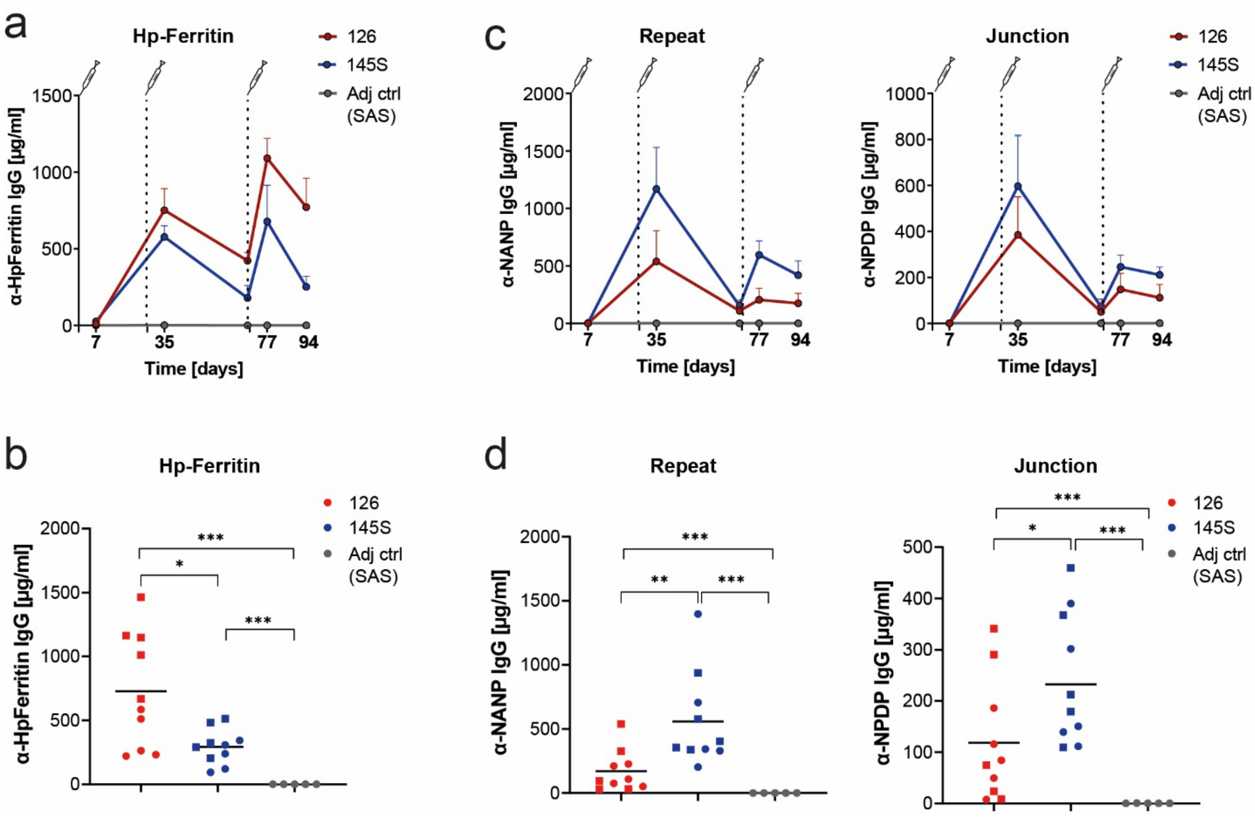

Suppl. Fig. 7 Glycan modifications of the nanoparticle focus the humoral response on PfCSP epitopes

(a) C57BL/6J mice were immunized with immunogen 126 or 145S adjuvanted with SAS at day 0, 28 and 70 and the serum IgG concentration at various timepoints against *H. pylori* -apoferritin was determined by ELISA. Immunization with adjuvants SAS alone served as control. One representative out of two independent experiments with 5 mice per group is shown.

(b) Comparison of the IgG concentration against *H. pylori*-apoferritin at day 94 after first immunization. Each dot represents one mouse. Pooled data of two independent

experiments with 5 mice per group are shown. Symbols indicate independent experiments.

(c) C57BL/6J mice were immunized with immunogen 126 or 145S adjuvanted with SAS at day 0, 28 and 70 and the serum IgG concentration at various timepoints against the repeat (NANP) and the junction (NPDP) was determined by ELISA. Immunization with adjuvant LMQ alone served as control. One representative out of two independent experiments with 5 mice per group is shown.

(d) Comparison of the IgG concentration against the repeat (NANP) and the junction (NPDP) at day 94 after first immunization. Each dot represents one mouse. Pooled data of two independent experiments with 5 mice per group are shown. Symbols indicate independent experiments. Arithmetic mean (a-d) and SEM (a,c) are indicated. Statistically-significant differences were calculated by two-tailed Mann–Whitney test (*P < 0.05; **P < 0.01; ***P < 0.001). Statistically non-significant differences are not indicated.

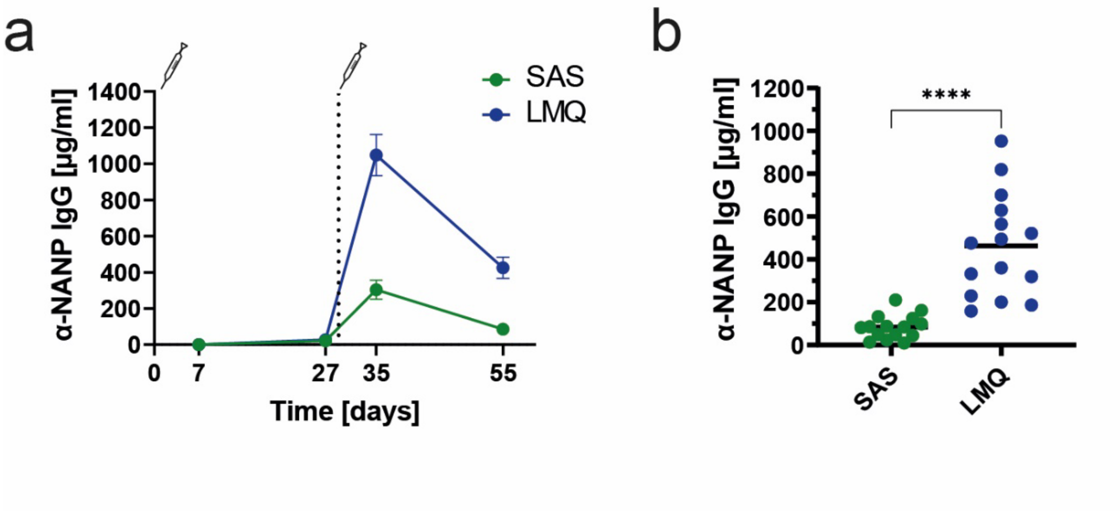

Suppl.

Suppl. Fig. 8 Adjuvants comparison for immunization with immunogen 145S

(a) C57BL/6J mice were immunized with immunogen 145S adjuvanted with SAS or LMQ at day 0 and 28 and the serum IgG concentration at various timepoints against the repeat (NANP) was determined by ELISA. One out of three independent experiments with 5 mice per group is shown.

(b) Comparison of the IgG concentration against repeat (NANP) at day 50 after first immunization. Each dot represents one mouse. Pooled data of the three independent experiments explained in (a) are shown (n=15).

Arithmetic mean (a,b) and SEM (a) are indicated. Statistically-significant differences were calculated by two-tailed Mann–Whitney test (****$P < 0.0001$).

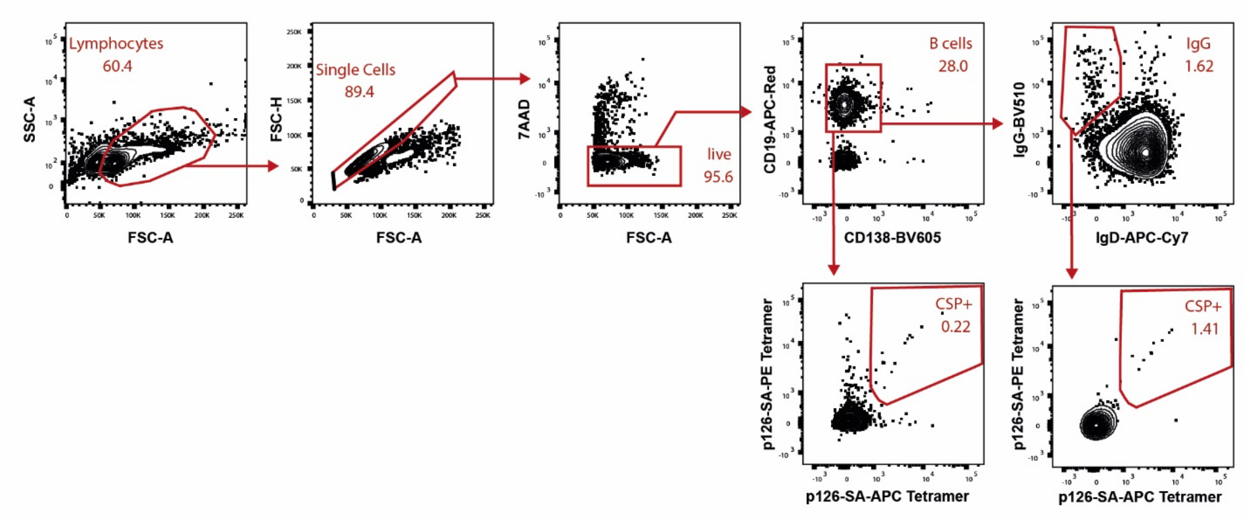

Suppl. Fig. 9 Gating strategy to detect IgG^{+}CSP^{+} cells (Fig. 3h)

Representative flow cytometric gating strategy for the detection of IgG^{+}CSP^{+} cells. Lymphocytes are gated by Forward and Sideward Scatter parameters (FSC-A and SSC-A), duplicates are excluded by size parameters (FSC-H and FSC-A), live cells are gated by exclusion of 7AAD^{+} cells. B cells are detected as CD19^{+}CD138^{-} cells, and IgG^{+} cells were defined as IgG^{+}IgD^{-} cells. CSP^{+} cells were detected by two color gating strategy as double-positive cells (p126-SA-APC and p126-SA-PE).

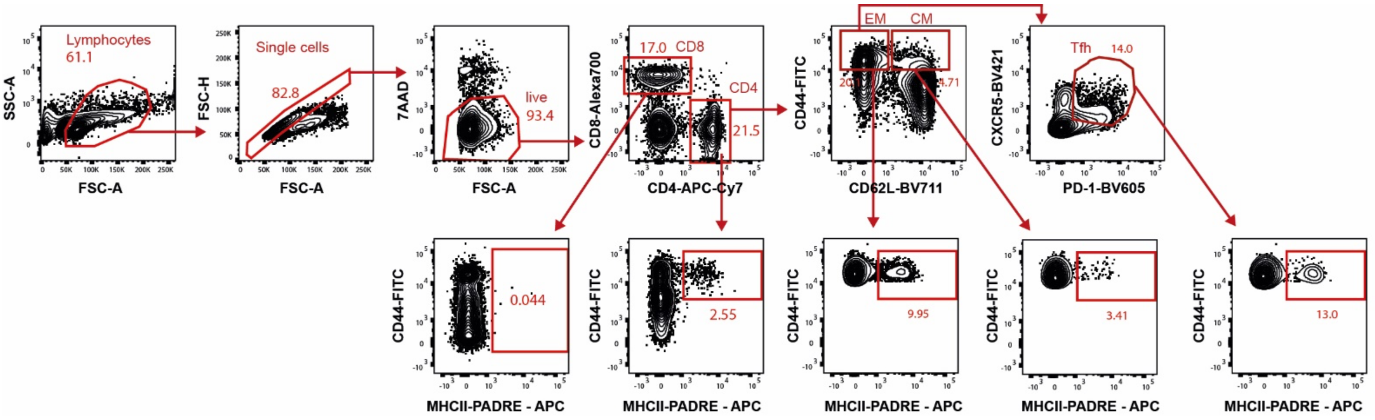

Suppl. Fig. 10 Gating of T cell subpopulations (Fig. 4a-c)

Representative flow cytometric gating strategy of T cell subpopulations *ex vivo*. Lymphocytes are gated by Forward and Sideward Scatter parameters (FSC-A and SSC-A), duplicates are excluded by size parameters (FSC-H and FSC-A), live cells are gating by the exclusion 7AAD$^+$ cells. Furthermore, T cells are detected as CD4$^+$ and CD8$^+$ T cells. Effector Memory (EM) and Central Memory (CM) T cells are gated as CD44^{high}CD62L$^-$ and CD44^{high}CD62L$^+$ respectively. T follicular helper cells (Tfh) were detected within the EM population as CXCR5$^+$PD-1$^+$ cells. PADRE$^+$ cells were detected by MHCII-Tetramer staining from different T cell populations.

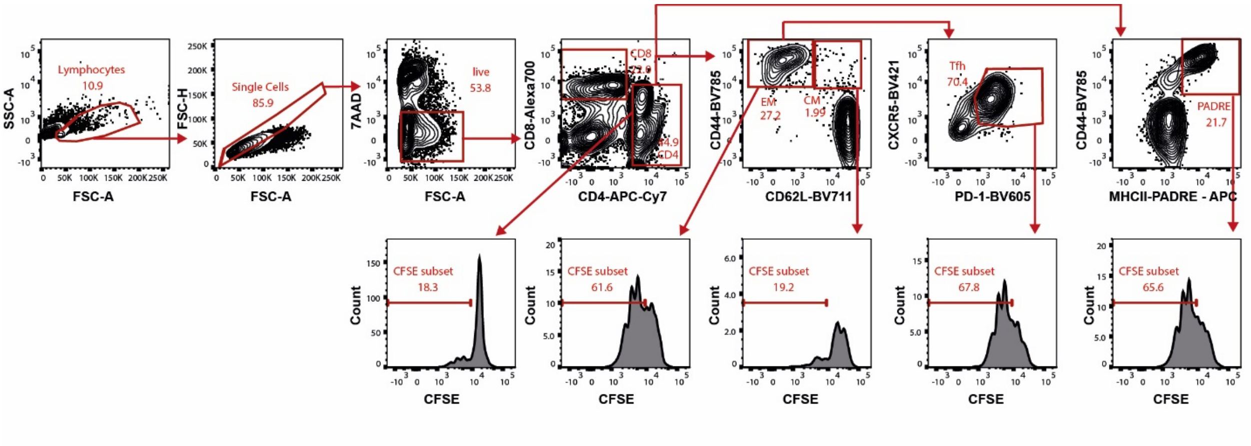

Suppl. Fig. 11 Gating of CFSE^{low} cells in cultured splenocytes (Fig. 4d-e)

Representative flow cytometric gating strategy of T cell subpopulations among *in vitro* cultured splenocytes. Lymphocytes are gated by Forward and Sideward Scatter parameters (FSC-A and SSC-A), duplicates are excluded by size parameters (FSC-H and FSC-A), live cells are gated by the exclusion 7AAD^{+} cells. T cells are detected as CD4^{+} and CD8^{+} T cells. Effector Memory (EM) and Central Memory (CM) T cells are gated as CD44^{high}CD62L^{-} and CD44^{high}CD62L^{+} respectively. T follicular helper cells (Tfh) were detected within the EM population as CXCR5^{+}PD-1^{+} cells. PADRE^{+} cells among CD4^{+} T cells were detected by MHCII-Tetramer staining. Dividing cells were gated as CFSE^{low} cells.

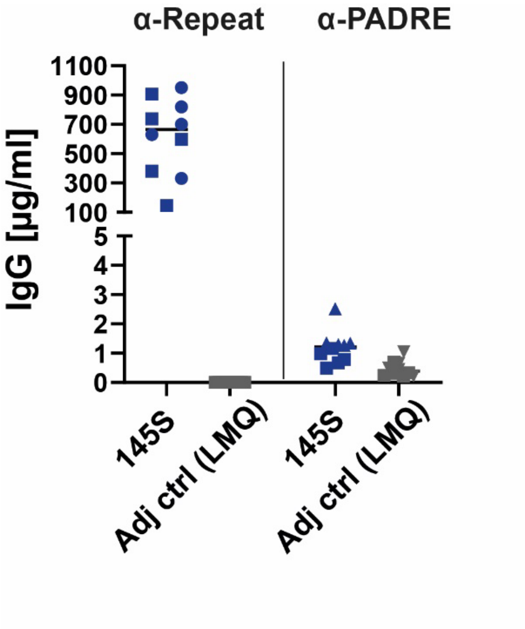

Suppl. Fig. 12 Comparison of the IgG concentration against PADRE and NANP

C57BL/6J mice were immunized with immunogen 145S adjuvanted with LMQ at day 0 and 28 and the serum IgG concentration at day 50 after the first immunization against the PADRE peptide and the repeat (NANP) was determined by ELISA. Dots represents individual mice. Arithmetic mean is indicated. Pooled data of two independent experiments with 5 mice per group are shown. Symbols indicate independent experiments.

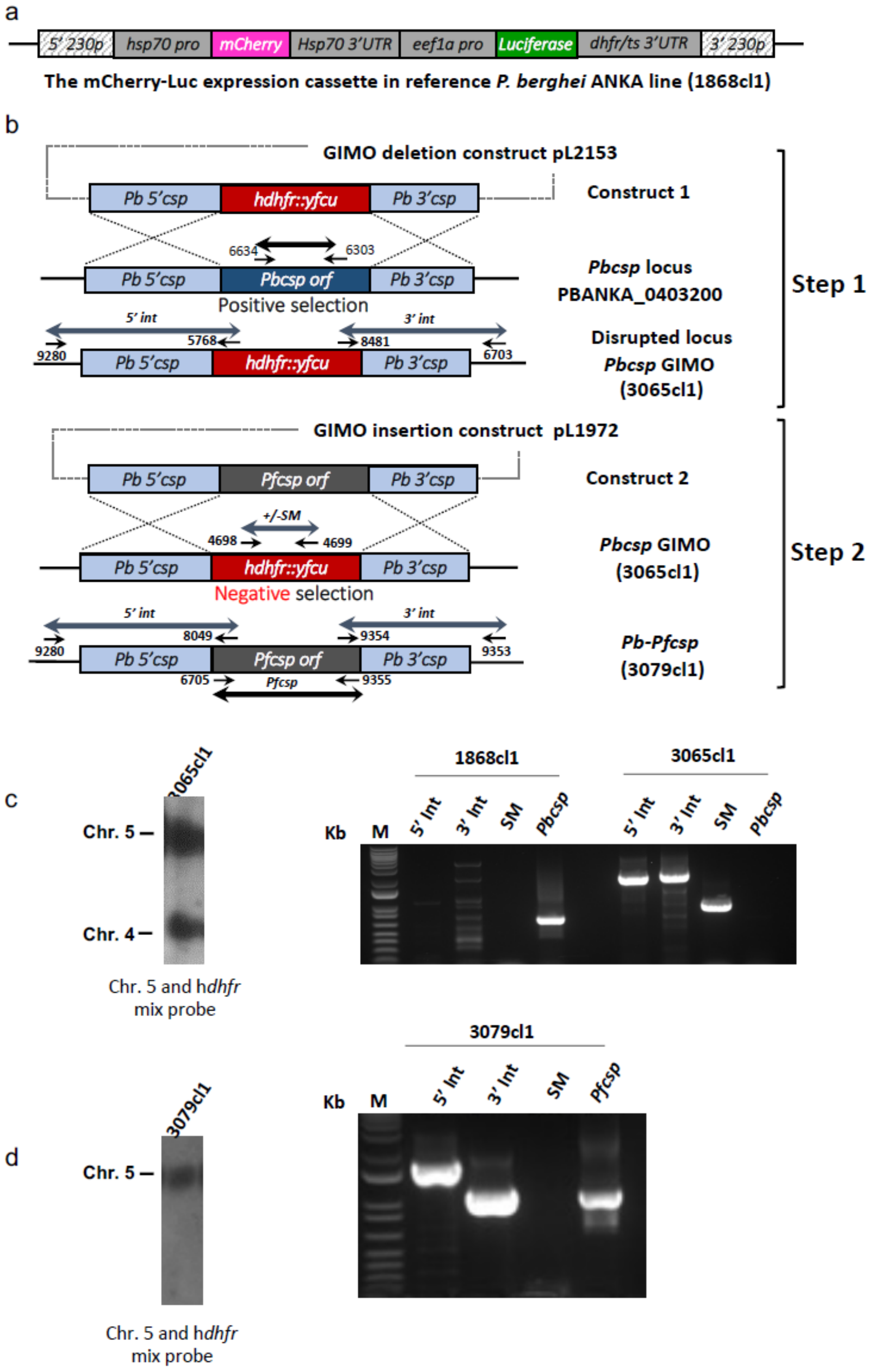

a
5' 230p
hsp70 pro
mCherry
Hsp70 3'UTR
eef1a pro
Luciferase
dhfr/ts 3'UTR
3' 230p
The mCherry-Luc expression cassette in reference P. berghei ANKA line (1868cl1)
b
GIMO deletion construct pL2153
Construct 1
Pb 5'csp
hdhfr::yfcu
Pb 3'csp
6634
6303
Pbcsp orf
Pbcsp locus
PBANKA_0403200
Positive selection
5' int
3' int
5768
8481
9280
6703
Disrupted locus
Pbcsp GIMO
(3065cl1)
Step 1
GIMO insertion construct pL1972
Construct 2
Pfcsp orf
+/-SM
4698
4699
Pbcsp GIMO
(3065cl1)
Step 2
Negative selection
8049
9354
9353
6705
9355
Pfcsp
Pb-Pfcsp
(3079cl1)
c
3065cl1
Chr. 5
Chr. 4
Chr. 5 and hdhfr
mix probe
1868cl1
3065cl1
Kb
M
5' Int
3' Int
SM
Pbcsp
d
3079cl1
Chr. 5
Chr. 5 and hdhfr
mix probe
Pfcsp

Suppl. Fig. 13 Generation and genotyping of the chimeric *P. berghei* line PbPfCSP(mCherry) **(line 3079cl1)**

Schematic representation of the generation of *Pb-Pfcsp* (line 3079cl1) using a 2-step gene insertion/marker out (GIMO) transfection protocol.

(a) Schematic representation of the Pb230p locus of the reference reporter *P. berghei* (ANKA) parasite line (1868cl1), containing mCherry and firefly luciferase reporter genes under the constitutive hsp70 and eef1a promoters introduced into the neutral p230p locus on chromosome 3. This line was used to generate the *Pb-Pfcsp* line.

(b) In the first step, plasmid pL2153 was used to introduce a selectable marker (SM) cassette into the *Pbcsp* locus (ORF, open reading frame) by double-crossover homologous recombination at the target regions (blue boxes). This plasmid contains the hdhf::yfcu SM cassette with hdhfr and yfcu under control of the eef1α promoter and the 3'UTR of Pbdhfr/ts. This plasmid was introduced in the reference reporter *P. berghei* line, 1868cl1 (see a). Positive selection with pyrimethamine after transfection results in selection of transgenic parasites where the *csp* gene is replaced by the hdhfr::yfcu, resulting in cloned line 3065cl1 (*Pb*-mCherry$_{hsp70}$-Luc$_{eef1a}$-*CSP*-GIMO). In the second step plasmid pL1972 was used to replace the positive-negative SM in the genome of the parasites of line 3065cl1 with the *Pfcsp* gene (PF3D7_0304600) by GIMO transfection. Plasmid pL1972 has the *Pfcsp* gene under the control of the *Pbcsp* 5' and 3' UTR regions. Negative selection with 5-FC and limiting dilution were used to select transgenic parasites where the hdhfr::yfcu SM in the *csp* locus is replaced by the *Pfcsp* gene (*Pb-Pfcsp* line). Cloning of the parasites resulted in line 3079cl1 (PbPfCSP(mCherry)).

(c) Genotyping of parasites of line 3065cl1 by Southern analysis and diagnostic PCR. Left: Southern analyses of pulsed field gel separated chromosomes of 3065cl1 confirmed the integration of the SM construct into the *Pbcsp* locus on chromosome (chr.) 4. Right: diagnostic PCR showing 5' and 3'-integration of construct pL2153, the presence of the SM and the absence of the *Pbcsp* ORF in line 3065cl1 and the absence of 5' and 3'-integration and SM and presence of Pb csp ORF in line 1868cl1.

(d) Genotyping of parasites of line 3079cl1 (PbPfCSP(mCherry)) by Southern analysis and diagnostic PCR. Left: Southern analyses of pulsed field gel separated chromosomes of 3079cl1 confirmed the deletion of the SM construct into the PbCSP locus on chr. 4. Right: diagnostic PCR showing 5' and 3'-integration of construct pL1972, the absence of the SM and the presence of the Pf*csp* ORF in line 3079cl1 (PbPfCSP(mCherry)). See Table S1 for primer sequences and expected sizes of PCR products.

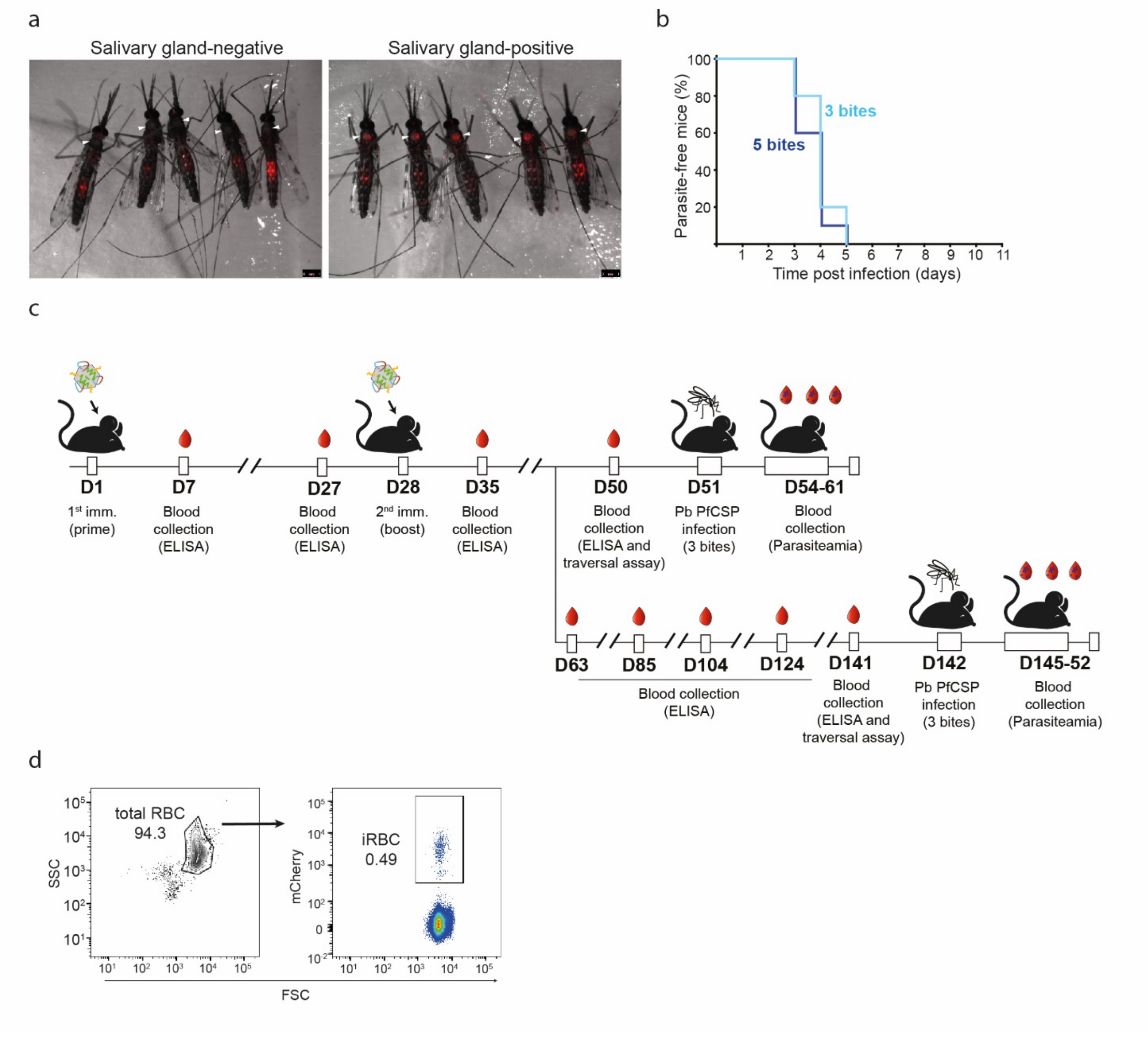

Suppl. Fig. 14 Challenge experiment set-up with mosquitoes infected with PbPfCSP(mCherry) **sporozoites**

(a) *A. gambiae 7b* line were infected with PbPfCSP(mCherry) parasites and 17 dpi mCherry signal (red) was detected under a fluorescence stereo microscope. Salivary gland-positive mosquitoes were selected for mice challenge procedures. Red signal is visible in the mosquito salivary gland areas (white arrows), abdomens (derived from midgut PbPfCSP(mCherry) oocysts infection) and eyes (derived from the *7b* transgenic

mosquito 3x*Pax3::RFP* reporter signal[57]). Representative pictures from 3 experiments, scale bar = 1 mm.

(b) C57BL/6J naïve mice (14 weeks old) were exposed to the bites of three (light blue) or five (dark blue) salivary gland-positive PbPfCSP(mCherry)-infected mosquitoes and parasitaemia was detected by FACS. Data show the percentage of parasite-free mice in one experiment with 10 mice per group.

(c) Scheme of prime-boost immunization adjuvanted with LMQ and timeline for the mosquito-bite challenge experiments.

(d) Representative flow cytometric gating strategy to detect parasitaemia during challenge experiments. Total red blood cells (RBC) are gated on FSC and SSC parameters, and infected RBC (iRBC) are detected as the mCherry-positive subpopulation.

Suppl. Table 1 Primers used in this study to generate and genotype transgenic parasites of PbPfCSP(mCherry)(line 3079cl1)

Primer code	Sequence	Product (kb)	Description
**Primers for genotyping of Pb-Pfcsp**			
9280	CAATTCCTCTTTCAATCTGTAC	2,3	5'integration pL2153
5768	CAGCGACGATGCAGTTTAGCGAACC		
8481	atgcGGATCCTAGGCCACACTACATGGTGAG	2,6	3'integration pL2153
6703	AGGTTGGTCATTGACACTCAGCGATATCATTACGACTTTGCTTAAAGG		
4698	GTTCGCTAAACTGCATCGTC	1,1	selectable marker cassette
4699	GTTTGAGGTAGCAAGTAGACG		
6634	GCATCCAAGCCCAAAGGAAC	0,8	Pb csp fragment
6303	CATTGAGACCATTCCTCTGTG		
9280	CAATTCCTCTTTCAATCTGTAC	1,7	5'integration p1972
8049	TAAGGCCTCAACAAATAAAA		
9354	GGTATTATCCTTCTTGTTCC	1,2	3'integration pL1972
9353	GTTACTATTCGTGCCCATTACGAC		
6705	ATGAGAAAATTAGCTATTTTATCTG	1,2	Pf csp
9355	GGAACAAGAAGGATAATACC		

Primers sequences and expected sizes of PCR products are indicated.
